# Supplementary material for: Economic evaluation of robot-assisted training versus an enhanced upper limb therapy programme or usual care for patients with moderate or severe upper limb functional limitation due to stroke: results from the RATULS randomised controlled trial
Source: BMJ Open. 2021 May 25;11(5):e042081. doi: 10.1136/bmjopen-2020-042081 (PMC8154983; doi:10.1136/bmjopen-2020-042081)
Supplement: Author's manuscript [file bmjopen-2020-042081.draft_revisions.pdf]

BMJ Open

BMJ Open is committed to open peer review. As part of this commitment we make the peer review history of every article we publish publicly available.

When an article is published we post the peer reviewers' comments and the authors' responses online. We also post the versions of the paper that were used during peer review. These are the versions that the peer review comments apply to.

The versions of the paper that follow are the versions that were submitted during the peer review process. They are not the versions of record or the final published versions. They should not be cited or distributed as the published version of this manuscript.

BMJ Open is an open access journal and the full, final, typeset and author-corrected version of record of the manuscript is available on our site with no access controls, subscription charges or pay-per-view fees (http://bmjopen.bmj.com).

If you have any questions on BMJ Open's open peer review process please email info.bmjopen@bmj.com

BMJ Open

Economic evaluation of robot-assisted training versus an enhanced upper limb therapy programme or usual care for patients with moderate or severe upper limb functional limitation due to stroke: results from the RATULS randomised controlled trial

Journal:	*BMJ Open*
Manuscript ID	bmjopen-2020-042081
Article Type:	Original research
Date Submitted by the Author:	25-Jun-2020
Complete List of Authors:	Fernandez-Garcia, Cristina; Newcastle University Faculty of Medical Sciences, Population Health Sciences Institute<br>Ternent, Laura; Newcastle University Faculty of Medical Sciences, Population Health Sciences Institute<br>Homer, Tara; Newcastle University Faculty of Medical Sciences, Population Health Sciences Institute<br>Rodgers, Helen; Newcastle University Faculty of Medical Sciences, Stroke Research Group - Population Health Sciences Institute; Northumbria Healthcare NHS Foundation Trust, Stroke Northumbria<br>Bosomworth, Helen; Newcastle University Faculty of Medical Sciences, Stroke Research Group, Population Health Sciences Institute<br>Shaw, Lisa; Newcastle University Faculty of Medical Sciences, Stroke Research Group, Population Health Sciences Institute<br>Aird, Lydia; Northumbria Healthcare NHS Foundation Trust, Stroke Northumbria<br>Andole, Sreeman; Barking Havering and Redbridge Hospitals NHS Trust<br>Cohen, David; London North West University Healthcare NHS Trust, Northwick Park<br>Dawson, Jesse; University of Glasgow, Institute of Cardiovascular and Medical Sciences<br>Finch, Tracy; Northumbria University, Nursing, Midwifery and Health<br>Ford, Gary; Oxford University Hospitals NHS Foundation Trust, Oxford Academic Health Science Network; Newcastle University Faculty of Medical Sciences, Stroke Research Group, Population Health Sciences<br>Francis, Richard; Newcastle University Faculty of Medical Sciences, Population Health Sciences Institute<br>Hogg, Steven ; Newcastle University Faculty of Medical Sciences, (Lay Investigator) contact Stroke Research Group, Population Health Sciences Institute<br>Hughes, Niall ; NHS Greater Glasgow and Clyde<br>Krebs, H; Massachusetts Institute of Technology,<br>Price, Christopher; Newcastle University Faculty of Medical Sciences, Stroke Research Group, Population Health Sciences; Northumbria Healthcare NHS Foundation Trust, Stroke Northumbria<br>Turner, Duncan; University of East London School of Health Sport and Bioscience<br>Van Wijck, Frederike; Glasgow Caledonian University, School of Health

	and Life Sciences<br>Wilkes, Scott; University of Sunderland, School of Pharmacy<br>Wilson, Nina; Newcastle University Faculty of Medical Sciences, Population Health Sciences Institute<br>Vale, Luke; Newcastle University Faculty of Medical Sciences, Population Health Sciences Institute
Keywords:	HEALTH ECONOMICS, STROKE MEDICINE, REHABILITATION MEDICINE

SCHOLARONE™ Manuscripts

I, the Submitting Author has the right to grant and does grant on behalf of all authors of the Work (as defined in the below author licence), an exclusive licence and/or a non-exclusive licence for contributions from authors who are: i) UK Crown employees; ii) where BMJ has agreed a CC-BY licence shall apply, and/or iii) in accordance with the terms applicable for US Federal Government officers or employees acting as part of their official duties; on a worldwide, perpetual, irrevocable, royalty-free basis to BMJ Publishing Group Ltd ("BMJ") its licensees and where the relevant Journal is co-owned by BMJ to the co-owners of the Journal, to publish the Work in this journal and any other BMJ products and to exploit all rights, as set out in our licence.

The Submitting Author accepts and understands that any supply made under these terms is made by BMJ to the Submitting Author unless you are acting as an employee on behalf of your employer or a postgraduate student of an affiliated institution which is paying any applicable article publishing charge ("APC") for Open Access articles. Where the Submitting Author wishes to make the Work available on an Open Access basis (and intends to pay the relevant APC), the terms of reuse of such Open Access shall be governed by a Creative Commons licence – details of these licences and which Creative Commons licence will apply to this Work are set out in our licence referred to above.

Other than as permitted in any relevant BMJ Author's Self Archiving Policies, I confirm this Work has not been accepted for publication elsewhere, is not being considered for publication elsewhere and does not duplicate material already published. I confirm all authors consent to publication of this Work and authorise the granting of this licence.

For peer review only

Economic evaluation of robot-assisted training versus an enhanced upper limb therapy programme or usual care for patients with moderate or severe upper limb functional limitation due to stroke: results from the RATULS randomised controlled trial

Cristina Fernandez-Garcia [1], Laura Ternent[1], Tara Homer[1], Helen Rodgers[2,3,4], Helen Bosomworth[2], Lisa Shaw[2], Lydia Aird[3], Sreeman Andole[5] , David L Cohen[6], Jesse Dawson[7] ,Tracy Finch[8], Gary A Ford[2,9], Richard Francis[1], Steven Hogg[10], Niall Hughes[11], Hermano I Krebs[12], Christopher I Price[2,3], Duncan L Turner[13], Frederike van Wijck[14], Scott Wilkes[15], Nina Wilson[1], and Luke Vale[1]

[1]Population Health Sciences Institute, Newcastle University, Newcastle upon Tyne, UK, [2]Stroke Research Group, Population Health Sciences Institute, Newcastle University, Newcastle upon Tyne, UK, [3]Stroke Northumbria, Northumbria Healthcare NHS Foundation Trust, North Tyneside, UK, [4]Newcastle Hospitals NHS Foundation Trust, Newcastle upon Tyne, UK, [5]Barking, Havering and Redbridge University Hospitals NHS Trust, Romford, UK,[6]London North West Healthcare NHS Trust, Northwick Park, UK, [7]Institute of Cardiovascular and Medical Sciences, University of Glasgow, Glasgow, UK, [8]Nursing, Midwifery & Health, Northumbria University, Newcastle upon Tyne, UK, [9]Oxford Academic Health Science Network, Oxford University Hospitals NHS Foundation Trust, Oxford, UK, [10]Lay investigator (contact Stroke Research Group, Population Health Sciences Institute, Newcastle University, Newcastle upon Tyne, UK), [11]NHS Greater Glasgow and Clyde, Glasgow, UK, [12]Massachusetts Institute of Technology, Cambridge, USA, [13]School of Health, Sport and Bioscience, University of East London, London, UK, [14]School of Health and Life Sciences, Glasgow Caledonian University, Glasgow, UK, [15]School of Medicine, University of Sunderland, Sunderland, UK.

Correspondence to Dr Laura Ternent laura.ternent@newcastle.ac.uk

Word count: 4,009

For peer review only - http://bmjopen.bmj.com/site/about/guidelines.xhtml

Abstract

Objective:

To determine whether robot-assisted training is cost-effective compared with an enhanced upper limb therapy (EULT) programme or usual care.

Design:

Economic evaluation within a randomised controlled trial.

Setting:

Four UK NHS centres.

Participants:

770 participants aged 18 or older with moderate or severe upper limb functional limitation from first-ever stroke.

Interventions:

Participants randomised to one of three programmes provided over a 12-week period: robot-assisted training plus usual care; the EULT programme plus usual care; or usual care.

Main economic outcome measures:

Mean healthcare resource use; costs to the NHS and personal social services in 2018 pounds; utility scores based on EQ-5D-5L responses; and quality-adjusted life years (QALYs). Cost-effectiveness reported as incremental cost per QALY and cost-effectiveness acceptability curves.

Results

At six months, on average usual care was the least costly option (£3,785) followed by EULT (£4,451) with robot-assisted training being the most costly (£5,387). Mean QALYs were highest for the EULT group (0.22) but no evidence of a difference was observed between the robot-assisted training (0.21) and usual care groups (0.21). The incremental cost per QALY at six months for participants randomised to EULT compared to usual care was £74,100. Cost-effectiveness acceptability curves showed that robot-assisted training was unlikely to be cost-effective and that EULT had a 19% chance of being cost-effective at the £20,000 willingness to pay (WTP) threshold. Usual care was most likely to be cost-effective at all the WTP values considered in the analysis.

Conclusions

The cost-effectiveness analysis suggested that neither robot-assisted training nor EULT, as delivered in this trial, were likely to be cost-effective at any of the cost per QALY thresholds considered.

Trial registration ISRCTN69371850

For peer review only - http://bmjopen.bmj.com/site/about/guidelines.xhtml

Strengths and limitations of this study

- This study fills a significant evidence gap being the first economic evaluation in the UK comparing robot-assisted training with an enhanced upper limb therapy (EULT) programme or usual care.
- Our economic evaluation was designed and conducted following best practice methods which resulted in robust and generalisable results.
- The unavailability of longer term data for the within trial evaluation means that no robust inferences could be made on the long-term cost-effectiveness of the interventions.

Introduction

Stroke is the fourth leading cause of death in the UK and a leading cause of disability with almost two thirds of stroke patients leaving hospital with a disability.(1) A common disability following a stroke is loss of upper limb function which reduces the individual's autonomy and impedes activities of daily living. Approximately 80% of people with acute stroke have upper limb motor impairment, with 50% of patients still experiencing problems after four years following the stroke.(2, 3)

The Robot-Assisted Training for the Upper Limb after Stroke (RATULS) trial sought to evaluate the clinical effectiveness and cost-effectiveness of robot-assisted training (using the MIT-Manus robotic gym system) by comparing it with either an enhanced upper limb therapy (EULT) programme, or usual care.(4, 5) Participants in the robot-assisted training and EULT groups also received usual care. The robot-assisted training and EULT programmes were of the same duration and frequency (45 minutes face-to-face therapy, three times a week for 12 weeks). Results from the trial provided no evidence that robot-assisted training as delivered in the study nor EULT improved upper limb function after stroke compared with usual care.(5)

After conducting a scoping review, we found little evidence of cost-effectiveness studies in the UK. The only economic evaluation we found in the literature assessed the cost-effectiveness of robot-assisted training therapy for upper limb rehabilitation within the US based VA Robotics study.(6, 7) This randomised controlled trial also assessed the cost-effectiveness the MIT-Manus robotic gym system in upper limb rehabilitation in stroke patients. However, differences in the healthcare system, in the configuration of the robotic gym and in the components of the comparison therapy meant that the results from this study lacked generalisability to the UK setting. This paper reports the results from a within-trial analysis that formed part of the RATULS trial.

Trial overview

Summary of RATULS Trial

The study was a three arm randomised controlled trial (RCT) which recruited 770 participants from stroke units, day hospitals, outpatient clinics, primary care, community rehabilitation services and

For peer review only - http://bmjopen.bmj.com/site/about/guidelines.xhtml

local stroke clubs in four study centres. The trial protocol(4) was approved by the National Research Ethics Committee Sunderland (reference 12/NE/0274).

Participants were eligible for inclusion in the study if they were 18 years or older, had experienced a first-ever stroke between one week and five years prior to randomisation and, as a consequence of the stroke, had moderate or severe upper limb functional limitation as measured by the Action Research Arm Test (ARAT)(8) (score 0-39). Potential participants who had been previously enrolled in this study; had participated or were participating in another upper limb rehabilitation study; had previously used the MIT-Manus robotic gym or another arm rehabilitation robot; had other notable upper limb impairment; or had a diagnosis that would interfere with the rehabilitation or outcome assessments were excluded from the trial. All those who were eligible and who consented to participate in the study were randomised on a 1:1:1 ratio to receive one of the three interventions.

The clinical primary outcome of the study was upper limb function "success" using (ARAT)(8) at three months post randomisation. Full details of the clinical trial results have been described elsewhere.(4, 5)

Economic evaluation methods

Perspective:

We conducted all analyses adopting the perspective of the UK National Health Service (NHS) and Personal Social Services (PSS) setting

Costs

The costs included in the analysis comprised the intervention costs for the robot-assisted training and EULT programmes, no intervention costs were directly associated with usual care and we assumed that any rehabilitation received had been reported in the health service utilisation questionnaire. We also included use of health and social services over the six month follow-up period for all randomised groups. For the intervention costs we assumed, as per the protocol,(4) that each therapy session in both intervention groups lasted 60 minutes including 45 minutes of face-to-face therapy on a 1:1 basis including the same staff component. In addition to staff time, capital costs were calculated for robot-assisted training using the "equivalent annual cost"(9) methodology applying a 3.5% discount rate and on the basis that the equipment would need replacing after five years.

We developed a health service utilisation questionnaire, informed by previous data collection tools including the Client Service Receipt Inventory (CSRI)(10) to capture health and social care resource use at baseline and six months post-randomisation. At baseline, participants were asked about the care received in the three months before they joined the study. These data were used to control for any imbalance in participant use of services at baseline. The information collected at six months included: visits to Accident & Emergency (A&E) departments; outpatient appointments; day patient appointments; overnight hospital stays; the use of GP and nursing services; use of therapy services; medications; community based healthcare; social services; and residential care and nursing home stays.

To estimate the cost at six months for each participant we combined data on use of care and services with unit costs obtained from routine data sources. We applied NHS national reference costs(11) to hospital based services. Information for staff costs, unit costs relating to primary care,

For peer review only - http://bmjopen.bmj.com/site/about/guidelines.xhtml

For peer review only

social care, and community health services was mostly derived from Curtis et.al. 2017.(12) In order to cost prescribed medication, we collected information on medication name, dosage, frequency and intake duration. We combined this information with unit costs taken from the British National Formulary.(13) When information on intake duration, medication format (e.g. dose; mode of intake) was missing, we used prescription cost analysis data(14) and assumed that the participant had been taking the most commonly prescribed format and had been issued one prescription for the trial period.

We used the information collected at six month follow-up in addition to the intervention costs to derive the total mean cost per participant per each randomised group. All costs are reported in pounds Sterling and converted to 2018 prices, when appropriate, using the Bank of England inflation calculator.(15)

Effectiveness outcome

The quality of life outcome measures used for the economic evaluation were the summary utility scores derived from the EQ-5D-5L questionnaires.(16) These were completed by participants at baseline, three and six months post-randomisation. The EQ-5D-5L questionnaire measures the individual's self-reported health-related quality of life through the following five domains: mobility, self-care, usual activities, pain/discomfort, and anxiety/depression. Respondents were asked to describe each domain according to five different levels: no problems, slight, moderate, severe, or extreme problems. Since there is no currently accepted valuation set for the EQ-5D-5L questionnaire, we mapped the questionnaire responses to the E5-5D-3L(17, 18) descriptive system in order to generate the utility values. These utility values formed the basis of our QALY calculations using the area-under-the-curve approach.(19)

Missing data

For the main (base-case) cost-effectiveness analysis we only included those participants for whom we had some data on costs. Once we determined that missing cost data were missing at random, we explored in the sensitivity analyses the effect on cost-effectiveness of applying multiple imputation to missing total costs controlling for age, sex and baseline ARAT score.

We explored the patterns of missing utility data. Once we established that information was missing at random, we used multiple imputation methods to estimate the missing utility values at three months and six months. This involved applying truncated normal regression whilst controlling for age, sex, and baseline ARAT score in order to generate the missing utility value.

Base-case cost-effectiveness analysis

We calculated costs and outcomes as mean values with standard deviations. Where we report differences in costs and effects between all three randomised groups we used 98.33% confidence intervals, as this was a three arm comparison. We conducted all pairwise comparisons using 95% confidence intervals. Using seemingly unrelated regression modelling methodology(20) in the adjusted cost-effectiveness analysis, we derived the incremental cost per gained QALY for each participant at six months. This approach involved estimating two linear regressions with their own dependent variable for costs and QALYs and a set of explanatory variables. We used randomised group, study centre, and time since stroke as explanatory variables for both costs and QALYs. Additionally, we incorporated baseline utility scores as an explanatory variable for the QALY equation and total baseline costs as an explanatory variable to the costs equation. We presented the results from the adjusted analysis in the form of an incremental cost-effectiveness ratio (ICER). The

For peer review only - http://bmjopen.bmj.com/site/about/guidelines.xhtml

ICER is the difference in costs divided by the difference in effects (in this case QALYs) between two alternatives.

In the analysis if a comparator was both more costly and less effective than the others it was dropped from any further cost-effectiveness comparisons because it was less cost-effective than the other comparator.

We created cost-effectiveness acceptability curves in order to assess the imprecision surrounding the estimates of costs, effects and cost-effectiveness. This approach involved drawing bootstrapped samples, with replacement, of the mean costs and mean QALYs from the original trial data. We repeated this process increasing the number of replications until the results were stable. This was achieved at 1,000 replications. After using the new values generated from the bootstrapping exercise to calculate the difference in costs and effects between groups, we combined this information with a range of willingness to pay (WTP) values (£0, £10,000, £20,000, £30,000, £50,000) per QALY gained. This generated a cost-effectiveness acceptability curve which graphically represented the probability of each of the interventions being cost-effective at each of the pre-specified value for society's WTP for a QALY.(21) All analyses were carried out in Stata 15.(22)

Sensitivity, subgroup and per-protocol analyses

We conducted deterministic sensitivity in order to assess the robustness of the cost-effectiveness results for three scenarios.

First, we examined the impact of assigning a value of zero to missing total healthcare costs.

Second, we examined the possibility that those participants with missing total healthcare costs may have used some services and hence incurred some costs. Under this scenario, once we established that information was missing at random, we applied truncated normal regression methods excluding total costs values below zero and above £25,000 (as the highest observed value for total costs) and used age, sex and baseline ARAT as covariates.

Finally, we investigated whether increasing the life span of the MIT-Manus robotic gym system from five to seven years would affect the cost-effectiveness of the interventions.

In order to explore the impact of time since stroke on the cost-effectiveness of the interventions, we conducted an exploratory analysis for the subgroups outlined in the protocol.(4) Three subgroups were pre-specified (< 3 months, 3-12 months and >12 months) according to time since stroke.

A secondary per-protocol cost-effectiveness analysis removing from the data set those participants who did not receive at least 20 sessions of therapy in the robot-assisted training and the EULT programme groups was also conducted. The cut-off point of 20 sessions was based on clinical evidence that an additional 20 hours of therapy compared with control interventions leads to improvements on functional outcome.(23)

We combined each sensitivity, subgroup and per-protocol analyses with bootstrapping in order to reflect the imprecision surrounding the cost-effectiveness results.

Extrapolation of trial results

We carried out a modelling exercise designed to extrapolate the mean QALYs at six months to 12 months based on the results of the trial, for this, we made a number of assumptions. First, we assumed that participants across all groups maintained the same utility levels reported at six months

For peer review only - http://bmjopen.bmj.com/site/about/guidelines.xhtml

208 post-randomisation. Second, we considered all intervention costs and therapy related costs (physiotherapy, occupational therapy, and speech and language therapy) as sunk costs, since they were deemed not to continue beyond the six month trial period. However, we assumed that all other levels of healthcare resource use remained constant from six to 12 months post randomisation.

We calculated the difference in mean costs and effects between the randomised groups with differences across groups not being formally tested. We used seemingly unrelated regression(20) methods to calculate the adjusted ICER at 12 months including the same explanatory variables used in the base-case cost-effectiveness analysis. As in the base-case analysis we used the bootstrapping technique to present uncertainty surrounding mean costs, effects, and cost-effectiveness.

Patient and Public Involvement

We have designed and reported our research with input from stroke survivors.

Results

Completeness of data

Most participants (96%) across all groups completed the health service utilisation questionnaire at baseline. There was a progressive increase in non-responses to the health service utilisation questionnaires over the six month follow up period. The pattern of responses was similar across the intervention groups however, the loss to follow-up was more pronounced in the usual care group. Whilst completion rates were 83% for the robot-assisted training group and 85% for the EULT group, this decreased to 70% of usual care participants. Completion rate of EQ-5D-5L at baseline was 99% across all of participants. This decreased to 88% at three months and 82% at six months. The highest number of non-responders belonged to the usual care group with response rates of 81% at three months and 75% at six months.

Health service use and costs.

Reported health service resource use was broadly similar across all randomised groups at six months (Table 1). Large standard deviations indicate that there was substantial variation in use of service between individuals in all three randomised groups, with a few participants reporting very high use of some services. Whilst not statistically tested, the main apparent difference between groups was in the therapy services received, with usual care being the heaviest user of home physiotherapy and speech and language therapy sessions compared with the robotic-assisted training and EULT groups. Participants in usual care had a higher reported mean number of contacts with general practice and nursing services compared with the robotic-assisted training and EULT groups.

Table 1- Reported health service resource use at six months

Area of resource utilisation	Robot-assisted training (n=257)		EULT (n=259)		Usual care (n=254)	
	Respondents n*	Mean contacts (SD)	Respondents n*	Mean (SD)	Respondents n*	Mean (SD)
GP surgery	205	1.80 (3.88)	208	1.49 (2.08)	168	1.83 (2.17)
GP home	209	0.37 (1.08)	213	0.27 (0.73)	174	0.38 (1.29)
GP phone	207	0.53 (1.22)	207	0.39 (0.96)	172	0.30 (0.77)
Nurse surgery	203	0.61 (1.39)	210	0.49 (1.19)	169	0.90 (5.58)

For peer review only - http://bmjopen.bmj.com/site/about/guidelines.xhtml

Nurse home	206	0.47 (2.07)	213	0.45 (1.65)	170	5.39 (44.63)
Nurse phone	211	0.08 (0.56)	212	0.52 (0.31)	174	0.04 (0.25)
NHS direct	211	0.11 (0.44)	213	0.08 (0.35)	174	0.03 (0.32)
Physiotherapy hospital	210	2.18 (6.10)	211	2.99 (9.23)	171	2.64 (7.72)
Physiotherapy home	205	2.76 (8.76)	207	3.10 (8.04)	170	4.35 (11.87)
Physiotherapy at general practice surgery	213	0.13 (1.16)	212	0.40 (2.63)	176	0.50 (3.85)
Physiotherapy elsewhere	212	0.23 (1.45)	213	0.21 (1.82)	175	0.62 (4.63)
Occupational therapy hospital	211	0.64 (3.14)	211	1.23 (7.23)	174	0.57 (3.53)
Occupational therapy home	210	1.56 (5.39)	209	1.27 (4.28)	173	1.95 (7.23)
Occupational therapy at general practice surgery	213	0.00 (0.07)	211	0.01 (0.14)	176	0.02 (0.23)
Occupational therapy elsewhere	212	0.16 (1.49)	211	0.00 (0.00)	176	0.00 (0.00)
Speech and language therapy hospital	213	0.57 (2.79)	213	0.65 (3.74)	173	0.94 (5.24)
Speech and language therapy home	210	0.47 (2.00)	212	0.64 (3.97)	175	2.22 (15.42)
Speech and language therapy at general practice surgery	212	0.00 (0.00)	213	0.03 (0.42)	176	0.02 (0.13)
Speech and language therapy elsewhere	213	0.05 (0.42)	213	0.47 (0.68)	175	0.03 (0.45)
A&E visits	213	0.33 (0.77)	214	0.37 (0.98)	178	0.24 (0.70)
Outpatient appointments	212	1.64 (3.14)	215	1.42 (2.88)	176	1.48 (4.24)
Hospital nights after being admitted via A&E	213	0.79 (4.77)	215	1.83 (12.95)	176	0.70 (3.59)
Hospital nights NOT admitted via A&E	213	0.28 (3.09)	215	0.03 (0.19)	176	0.25 (1.81)
Day patient treatment (half day)	205	0.08 (0.35)	210	0.09 (0.46)	175	0.06 (0.32)
Day patient treatment (full day)	199	0.03 (0.17)	201	0.02 (0.18)	169	0.06 (0.07)
Residential care	213	1.75 (14.65)	215	2.08 (17.07)	177	1.39 (10.72)
Nursing home	213	0.00 (0.00)	216	0.83 (12.24)	177	3.08 (23.51)
Meals on wheels	213	0.02 (0.27)	213	0.04 (0.49)	177	0.08 (1.05)
Home help personal care	211	2.89 (6.77)	210	3.04 (7.42)	173	3.17 (7.03)
Home help household tasks	213	0.74 (3.21)	212	0.68 (3.87)	175	1.06 (4.54)
Home help shopping	213	0.11 (0.74)	212	0.07 (0.53)	176	0.11 (0.76)
Health visitor	212	0.05 (0.45)	213	0.09 (0.85)	177	0.03 (0.28)
Geriatrician	212	0.03 (0.42)	213	0.02 (0.23)	177	0.00 (0.00)
Psychiatrist	212	0.13 (0.72)	213	0.75 (0.54)	175	0.01 (0.11)
Psychologist	209	0.45 (2.07)	211	0.28 (1.14)	176	0.44 (2.37)
Chiropodist	210	0.55 (1.19)	210	0.39 (1.06)	173	0.44 (1.02)
Optician	210	0.25 (0.56)	212	0.23 (0.53)	174	0.32 (0.64)
Pharmacist	211	0.66 (2.33)	207	0.60 (2.28)	174	1.16 (4.35)

For peer review only - http://bmjopen.bmj.com/site/about/guidelines.xhtml

n*= Number of participants who completed all or part of the questionnaire<br>SD: Standard deviation; GP: General practitioner; A&E: Accident and Emergency

The mean total cost per participant for each randomised group is reported in Table 2. We also report the mean total cost per cost category per participant. The highest mean costs per participant were associated with the use of social care services which included stays in residential and nursing home facilities and care assistance received at home. The average cost per participant was higher in the usual care group in all categories except in secondary care and other NHS and social services used by the participants. The addition of the intervention costs however, reversed this finding making the mean cost per participant highest in the group receiving robot-assisted training. We found some evidence the mean difference in costs between the robot-assisted training group and the usual care group was higher (Mean difference: 1,601 [95% CI 706 to 2,496]). However, there was no evidence of a difference between the EULT group and usual care higher (Mean difference: 665 [95% CI -444 to 1,774]).

Table 2 – Total cost (£) over six months for all participants with full economic data

**Area of resource utilisation**	**Robot-assisted training (n= 257)**		**EULT (n=259)**		**Usual care (n=254)**	
	n	Mean (SD)	n	Mean (SD)	n	Mean (SD)
Intervention costs	257	2,872 (0)	259	1,399 (0)	0	-(-)
Primary care costs and community based health care (including therapy services)	213	743 (1,031)	215	777 (1,262)	177	1,078 (1,813)
Social care	213	1,410 (3,146)	216	1,541 (3,943)	178	1,890 (4,281)
Secondary care	213	733 (2,247)	216	988 (4,486)	178	668 (1,880)
Medication costs	157	149 (302)	162	154 (273)	126	198 (347)
Other NHS and social services	11	727 (983)	13	790 (946)	9	307 (406)
Deceased participants	1	0 (0)	3	13,953 (4,516)	0	- (-)
Mean total cost	257	5,387 (4,054)	259	4,451 (6,033)	178	3,785 (5,437)
Mean difference between robot-assisted training and usual care with 95% confidence interval	1,601 [706 to 2,496]					
Mean difference between EULT and usual care with 95% confidence interval	665 [-444 to 1,774]					
SD: Standard deviation;<br>n= randomised n						

Health outcomes.

The mean utility scores across all randomised groups were similar at baseline, three months and six months as were mean QALYs (Table 3). The EULT group had the highest mean QALY (0.23) followed by robot-assisted training and usual care groups, both of which had a mean QALY over six months of

For peer review only - http://bmjopen.bmj.com/site/about/guidelines.xhtml

260 0.21. The mean differences in QALYs between each of the intervention groups (robot-assisted training and EULT) and usual care were found to be very small and there was no evidence of a difference between randomised groups.

263 *Table 3 - Utility scores at baseline, three months and six months and QALYs at six months*

Time period	Robot-assisted training (n*=257)		EULT (n*=259)		Usual care (n*=254)	
	n	Mean (SD)	n	Mean (SD)	n	Mean (SD)
Baseline EQ-5D-5L score	254	0.36 (0.26)	259	0.39 (0.25)	254	0.37 (0.26)
3 month EQ-5D-5L score	232	0.45 (0.27)	236	0.48 (0.24)	207	0.42 (0.29)
6 month EQ-5D-5L score	223	0.46 (0.29)	222	0.50 (0.27)	190	0.46 (0.27)
QALYs at 6 months after multiple imputation	254	0.21 (0.12)	259	0.23 (0.10)	254	0.21 (0.11)
Mean difference in QALYs between robot-assisted training and usual care with 95% confidence interval	0.00 (-0.20 to 0.20)					
Mean difference in QALYs between EULT and usual care with 95% confidence interval	0.02 (0.00 to 0.35)					
n*= number randomised; SD: Standard deviation; QALY: Quality adjusted life year						

264

265 *Cost-effectiveness analysis, subgroup analysis, per-protocol analysis and longer term model.*

266 The base-case cost-effectiveness analysis results (Table 4) show the adjusted ICER was £74,100 for the comparison between EULT and usual care. Robot-assisted therapy was, on average, dominated by EULT since it was both, on average, more costly and less effective. The cost-effectiveness acceptability curve (Figure 1), shows that EULT had a 19% chance of being cost-effective at the £20,000 WTP threshold value. The probability of EULT being cost-effective remained below 40% at all the WTP values considered in the analysis. Robot-assisted training had no probability of being cost-effective at all WTP values considered in the analysis.

273 The results from the subgroup analysis are also summarised in Table 4. These showed that robot-assisted training remained dominated on average by EULT in both the adjusted and unadjusted results for all subgroups. The highest ICER (£126,143) comparing EULT with usual care was linked to those participants who had a stroke more than 12 months before randomisation. The subgroup of participants who were less than three months post-stroke at randomisation had the lowest ICER (£31,400) for the comparison of EULT with usual care. The bootstrapped sensitivity analysis for this group suggested that EULT had a 41% probability of being cost-effective at the £20,000 WTP threshold.

For peer review only - http://bmjopen.bmj.com/site/about/guidelines.xhtml

For peer review only

Results from the per-protocol analysis did not change the direction of the cost-effectiveness results. Usual care remained the least costly option followed by EULT and robot-assisted training. The ICER for EULT and usual care was £68,000 and EULT only had a 17% probability of being cost effective at the £20,000 WTP threshold. The probability of robot-assisted therapy being considered cost-effective was very low.

Table 4 shows the results from the economic model which extrapolated the trial data on costs and effects to 12 months. Unadjusted mean costs per participant were lowest in the EULT group (£6,892) followed closely by usual care (£6,916) and robot-assisted training (£7,538). Mean QALYs were in line with those seen in the base-case analysis with participants in the EULT group having highest mean QALYs at 12-months (0.48) followed by usual care (0.47) and robot-assisted training (0.44). Once we adjusted for baseline costs, baseline utility score, study centre, randomised group and time since stroke, usual care reverted to being the least costly option. The ICER for the comparison between EULT and usual care was £6,095, however there was only a 55% probability of EULT being considered cost-effective compared with usual care at the £20,000 WTP value. Robot-assisted training had no probability of being cost-effective at this WTP value.

Table 4 - Results from base-case cost-effectiveness analysis, subgroup analyses and longer term economic model

Scenario	Robot-assisted training (n=257)	EULT (n=259)	Usual care (n=254)	ICER	Probability of each therapy being cost-effective at the £20,000 WTP threshold		
					Robot-assisted training	EULT	Usual care
Base-case analysis							
Cost – £, unadjusted, mean (CI)*	5,387 (4,777 to 5,996)	4,451 (3,548 to 5,354)	5,387 (4,777 to 5,996)	-	-	-	-
QALY - unadjusted, mean (CI)*	0.21 (0.195 to 0.229)	0.23 (0.213 to 0.244)	0.21 (0.194 to 0.230)	-	-	-	-
ICER (£ per QALY) – adjusted EULT vs Usual care§	-	-	-	74,100	0.00	0.19	0.81
Subgroup analysis (Less than 3 months - time since stroke)							
Cost – £, unadjusted, mean (CI)	5,485 (3,938 to 7,032)	3,863 (2,527 to 5,199)	3,328 (1,443 to 5,213)	-	-	-	-
QALY - unadjusted, mean (CI)*	0.22 (0.19 to 0.25)	0.24 (0.20 to 0.28)	0.21 (0.17 to 0.25)	-	-	-	-
ICER (£ per QALY) – adjusted EULT vs Usual care§	-	-	-	31,400	0.00	0.41	0.59
Subgroup analysis (3 to 12 months - time since stroke)							
Cost – £, unadjusted, mean (CI)*	5,790 (4,793 to 6,786)	5,084 (3,393 to 6,774)	4,943 (3,228 to 6,658)	-	-	-	-
QALY - unadjusted, mean (CI)	0.20 (0.18 to 0.23)	0.23 (0.20 to 0.25)	0.21 (0.19 to 0.24)	-	-	-	-

For peer review only - http://bmjopen.bmj.com/site/about/guidelines.xhtml

ICER (£ per QALY) – adjusted EULT vs Usual care§	-	-	-	79,400	0.04	0.37	0.59
Subgroup analysis (More than 12 months)							
Cost – £, unadjusted, mean (CI)*	4,822 (4,036 to 5,728)	3,961 (2,783 to 5,138)	2,823 (1,299 to 4,348)	-	-	-	-
QALY - unadjusted, mean (CI)*	0.22 [0.18 to 0.25]	0.23 [0.20 to 0.25]	0.21 (0.18 to 0.24]	-	-	-	-
ICER (£ per QALY) – adjusted EULT vs Usual care§	-	-	-	126,143	0.01	0.15	0.84
Per-protocol analysis							
Cost – £, unadjusted, mean (CI)*	5,595 (4,929 to 6,261)	4,551 (3,596 to 5,501)	3,785 (2801 to 4770)	-	-	-	-
QALY - unadjusted, mean (CI)*	0.22 (0.20 to 0.24)	0.23 (0.21 to 0.25)	0.21 (0.19 to 0.23)	-	-	-	-
ICER (£ per QALY) – adjusted EULT vs UC§	-	-	-	68,000	0.00	0.17	0.83
Extrapolation to 12 months							
Cost – £, unadjusted, mean (CI)*	7,538 (6,350 to 8,725)	6,892 (5,149 to 8,635)	6,916 (5,003 to 8,830)	-	-	-	-
QALY - unadjusted, mean (CI)*	0.44 (0.40 to 0.48)	0.48 (0.44 to 0.51)	0.45 (0.41 to 0.48)	-	-	-	-
ICER (£ per QALY) – adjusted EULT vs Usual care§	-	-	-	6,095	0.10	0.55	0.35

QALY: quality adjusted life year
CI: confidence interval
ICER: Incremental cost-effectiveness ratio
WTP: Willingness to pay
* 98.33% confident interval used throughout the analyses for the three arm comparison
§ data adjusted for baseline costs, baseline utility score, study centre, randomised group and time since stroke

(Insert Figure 1)

Sensitivity analyses

The different scenarios explored in these analyses did not change the direction of the results from the base-case cost-effectiveness results. Robot-assisted training remained dominated on average by EULT in all instances. First, when we changed the missing costs to zero, the resulting ICER between EULT and usual care increased to £172,000. This increase is to be expected since all participants with missing total costs belong to the usual care group. By imputing zero for missing costs the mean costs

For peer review only - http://bmjopen.bmj.com/site/about/guidelines.xhtml

for the usual care participants decreased and hence, when this was done, the ICER increased when compared to EULT.

Second, applying multiple imputation methods to missing costs resulted in an increase to the unadjusted mean usual care costs (£4,451) compared with the base-case results (£3,785). Consequently, the resulting ICER from the comparison between EULT and usual care decreased to £50,000 with the probability of EULT being cost-effective at £20,000 increasing to 27%.

Third, extending the life of the robotic gym system resulted in a reduction of the mean capital costs per patient and hence, in a lower mean total cost for the robot-assisted training group (£5,085) compared with the base-case analysis (£5,387). There were no changes to the utility scores across all groups nor to the mean costs for EULT and usual care, hence the resulting ICER for the comparison of EULT and usual care remained the same as in the base-case analysis (£74,100).

Discussion

The RATULS trial found no evidence that robot-assisted training, as delivered in the study, improved upper limb function "success" for stroke patients with moderate or severe upper limb functional limitation when compared with a EULT programme or usual care.(5) Our economic evaluation strengthens the evidence base of these upper limb rehabilitation programmes through the evaluation of their cost-effectiveness.

Results from the base-case cost-effectiveness analysis suggested that, on average, robot-assisted training was more costly than both EULT and usual care and that robot-assisted training was slightly less effective than EULT. EULT was on average more costly and as effective as usual care in the unadjusted analyses and more costly and more effective in the adjusted analyses. The balance of probabilities favoured usual care as the preferred upper limb rehabilitation therapy over the range of WTP values considered. Focusing on the society's WTP for a QALY, the bootstrapped analysis suggested that, EULT, as delivered in this trial, was unlikely to be cost-effective over any of the WTP values considered, despite being more effective than robot-assisted training and usual care. The subgroup, sensitivity and per-protocol analyses did not change the direction of the base-case cost-effectiveness results. Extrapolating within-trial results to 12 months produced the lowest ICER for the comparison between EULT and usual care overall, however, high uncertainty surrounds the assumptions made about how costs and utilities change beyond the trial follow-up.

The main strength of this economic evaluation is that it was conducted as part of a rigorously run RCT and followed guidelines for best practice throughout.(24, 25) As a result, we were able to base the economic evaluation on individual patient data collected during the trial and benefited from low levels of missing healthcare resource use and quality of life data. However, the loss to follow up in the usual care group may have led to an underestimation of resource use for these participants. Nevertheless, when we imputed missing values the results were still consistent with those from the base-case analysis.

Through the collection of self-reported quality of life information at three points during the study using the EQ-5D-5L questionnaire,(16) we were able to measure quality of life gains for participants across all groups. One strength of this generic tool is that decision makers will be able to make priority-setting decisions not only for this patient group but across different disease areas.(25)

A noteworthy limitation of the economic evaluation is associated with the timeframe of the trial. The within-trial economic evaluation assessed the cost-effectiveness of the interventions at six months.

For peer review only - http://bmjopen.bmj.com/site/about/guidelines.xhtml

For peer review only

A longer term perspective was originally planned but due to limitations of the data, extrapolation to 12 months only was conducted. The results however, need to be interpreted with caution due to the assumptions made on both costs and utility values.

The economic evaluation fills a significant evidence gap with this being the first economic evaluation comparing robot-assisted training with usual care having been conducted in the UK NHS setting. This evaluation expands the analysis conducted on the cost-effectiveness of the MIT-Manus robotic system as part of the VA Robotics trial.(6, 7) This study, confined to the US healthcare system, reported a small QALY gain for the robot group compared to usual care and not significant differences in costs between groups. Differences in the healthcare system between both countries means that our economic evaluation is key for making cost-effectiveness results relevant to the UK NHS setting. Furthermore, it takes into account a number of key differences in the design of both studies. First, whilst the VA robotics trial assumed that the robotic gym could be used simultaneously by two patients, the RATULS trial was designed to deliver robot-assisted training on a one-to-one basis. Second, the components of the intensive comparison therapy differed in each study. Third, we used E5-5D-5L as the recommended tool to calculate QALYs, whilst the VA robotic trial calculated QALYs from responses to the Health Utility Index Mark 3 (HUI3) questionnaire.(26) All these points reduce the grounds for comparability between studies and supports the need for the economic analysis we conducted alongside the RATULS trial.

The use of multiple sites contributed to the generalisability of the economic evaluation. The analyses controlled for differences in sites hence minimising the chance of obtaining biased results from differences in costs and effects driven by location.

In conclusion, robot-assisted training was not found to be cost-effective in comparison to EULT and usual care. This economic evaluation suggested that usual care remained the most cost-effective type of upper limb rehabilitation compared with a EULT therapy programme and robot-assisted training for stroke patients with moderate or severe functional limitation.
The results create opportunities for further research. In particular, further research could explore the potential effect on both costs and QALYs from reconfigurations to the delivery of EULT and robot-assisted training. It remains unclear, for example, whether delivering therapy in a group or classroom setting may first, be feasible in the NHS setting and second improve the quality of life and clinical outcomes. Further development of these interventions may increase their cost-effectiveness compared to usual care. Additionally, studies with a longer follow-up data may help establish whether the QALY gains derived from the interventions are sustained beyond the set timeframe of the trial, this can then lead to a robust assessment of their long-term cost-effectiveness.

Figure 1 - Cost-effectiveness acceptability curve (base-case analysis) - adjusted bootstrapped replications for cost-effectiveness analysis

For peer review only - http://bmjopen.bmj.com/site/about/guidelines.xhtml

Contributors:

CFG conducted the health economic evaluation. LV and LT are senior health economists and were involved in the main study design, delivery, analysis and interpretation of the data and drafting the manuscript. TH contributed to the health economic evaluation and drafting of the manuscript. HR was the Chief Investigator of the study. HB, HIK, FvW, DH, GAF, LS, LA, TF, CIP, DLT, SW, JD, were involved in the main study design, delivery, data management, data analysis and drafting the manuscript. NW, NA, SA, DLC, RF, NH contributed to study delivery, interpretation of the data and drafting the manuscript. SH was a co-investigator and is a service user, he was involved in the main study design and delivery, interpretation of data. All authors have commented upon drafts of the manuscript and have given final approval to this version.

Acknowledgements:

We would like to thank Jenni Hislop for her input into the health economics analysis plan and the development of the data collection tools.

Funding statement:

This work was supported by the National Institute for Health Research Health Technology Assessment Programme (reference: 11/26/05). The views and opinions expressed here are those of the authors and do not necessarily reflect those of the HTA programme, NIHR, the UK National Health Service (NHS), or UK Department of Health.

Data availability statement:

De-identified participant data will be made available to scientific researchers upon approval of their study protocol and analysis plan, by a committee of the RATULS team. Proposals should be directed to the corresponding author. A data sharing agreement will need to be signed by data requestors. Requests for data sharing will be considered after publication of the full trial report in the Health Technology Assessment Journal which is anticipated in 2020.

Competing interests:

Professor Rodgers reports grants from NIHR, during the conduct of the study; personal fees from Bayer, outside the submitted work; and Member of NIHR HTA CET panel 2010 to 2014. Professor Ford reports grants from National Institute for Health Research, during the conduct of the study; personal fees from Amgen, personal fees from Daiichi Sankyo, grants and personal fees from Medtronic, personal fees from Stryker, personal fees from Pfizer, personal fees from Bayer, outside the submitted work. Professor Price and Dr Lisa Shaw report grants from National Institute for Health Research, during the conduct of the study. Professor Krebs reports other from Interactive Motion Technologies, other from 4Motion Robotics, outside the submitted work; in addition, Professor Krebs has a patent Interactive Robotic Therapist; US Patent 5,466,213; 1995; Massachusetts Institute of Technology issued, and a patent Wrist And Upper Extremity Motion; US Patent No. 7,618,381; 2009; Massachusetts Institute of Technology licensed to Bionik Laboratories.

For peer review only

For peer review only - http://bmjopen.bmj.com/site/about/guidelines.xhtml

References

1. Stroke Association, UK. State of the nation stroke statistics - February 2018. Available from: https://www.stroke.org.uk/system/files/sotn_2018.pdf (accessed 15th September 2018).

2. Langhorne P, Coupar F, Pollock A. Motor recovery after stroke: a systematic review. The Lancet Neurology. 2009;8:741-54.

3. Broeks J, Lankhorst G, Rumping K, et al. The long-term outcome of arm function after stroke: results of a follow-up study. Disability and Rehabilitation. 1999;21(8):357-64.

4. Rodgers H, Shaw L, Bosomworth H, et al. Robot assisted training for the upper limb after stroke (RATULS): study protocol for a randomised controlled trial. Trials. 2017;18:340.

5. Rodgers H, Bosomworth H, Krebs HI, et al. Robot assisted training for the upper limb after stroke (RATULS): a multicentre randomised controlled trial. The Lancet. 2019;394:51-62.

6. Wagner TH, Lo AC, Peduzzi P, et al. An economic analysis of robot-assisted therapy for long-term upper-limb impairment after stroke. Stroke. 2011;42(9):2630-2.

7. Lo AC, Guarino P, Krebs HI, et al. Multicenter randomized trial of robot-assisted rehabilitation for chronic stroke: methods and entry characteristics for VA ROBOTICS. Neurorehabilitation and Neural Repair. 2009;23(8):775-83.

8. Lyle RC. A performance test for assessment of upper limb function in physical rehabilitation treatment and research. International Journal of Rehabilitation Research. 1981;4(4):483-92.

9. Drummond MF, Sculpher MJ, Torrance GW, et al. Methods for the economic evaluation of health care programmes. Oxford University Press; 2005.

10. Beecham J, Knapp M. Client Service Receipt Inventory (CSRI). Database of Instruments for Resource Use Management. 1992.

11. NHS Improvement. NHS Reference costs 2017/2018 London: Department of Health; 2018. Available from: https://improvement.nhs.uk/resources/reference-costs (accessed 1st September 2018).

12. Curtis L, Burns, Amanda. Unit Costs of Health & Social Care 2017. PSSRU - University of Kent. 2017.

13. British national formulary (BNF) 75: Joint Formulary Committee; 2018. Available from: www.medicinescomplete.com (accessed 1st November 2018).

14. Prescription Cost Analysis - England, 2017 NHS Digital; 2018; cited NHS Digital. Available from: https://digital.nhs.uk/data-and-information/publications/statistical/prescription-cost-analysis/prescription-cost-analysis-england-2017 (accessed 1st November 2018).

For peer review only - http://bmjopen.bmj.com/site/about/guidelines.xhtml

15. Bank of England Inflation Calculator 2018. Available from: https://www.bankofengland.co.uk/monetary-policy/inflation/inflation-calculator (accessed 1st November 2018).

16. Williams A. The EuroQol instrument. EQ-5D concepts and methods: a developmental history: Springer; 2005. p. 1-17.

17. NICE. Position statement on use of the EQ-5D-5L valuation set. 2017.

18. Van Hout B, Janssen M, Feng Y-S, et al. Interim scoring for the EQ-5D-5L: mapping the EQ-5D-5L to EQ-5D-3L value sets. Value in Health. 2012;15(5):708-15.

19. Billingham L, Abrams KR, Jones DR. Methods for the analysis of quality-of-life and survival data in health technology assessment. 1999.

20. Willan AR, Briggs AH, Hoch JS. Regression methods for covariate adjustment and subgroup analysis for non-censored cost-effectiveness data. Health Economics. 2004;13(5):461-75.

21. Fenwick E, Claxton K, Sculpher M. Representing uncertainty: the role of cost-effectiveness acceptability curves. Health Economics. 2001;10(8):779-87.

22. StataCorp. Stata Statistical Software: Release 15. College Station, TX: StataCorp LLC.; 2017.

23. Pollock A, Farmer SE, Brady MC, et al. Interventions for improving upper limb function after stroke. Cochrane Database of Systematic Reviews. 2014(11).

24. NICE. Developing NICE guidelines: the manual: chapter 4 - incorporating economic evaluation. National Institute for Health and Care Excellence Manchester, England; 2014.

25. NICE. Guide to the methods of technology appraisal 2013. National Institute for Health and Care Excellence; 2013.

26. Feeny D, Furlong W, Torrance GW, et al. Multiattribute and single-attribute utility functions for the health utilities index mark 3 system. Medical Care. 2002;40(2):113-28.

For peer review only

For peer review only - http://bmjopen.bmj.com/site/about/guidelines.xhtml

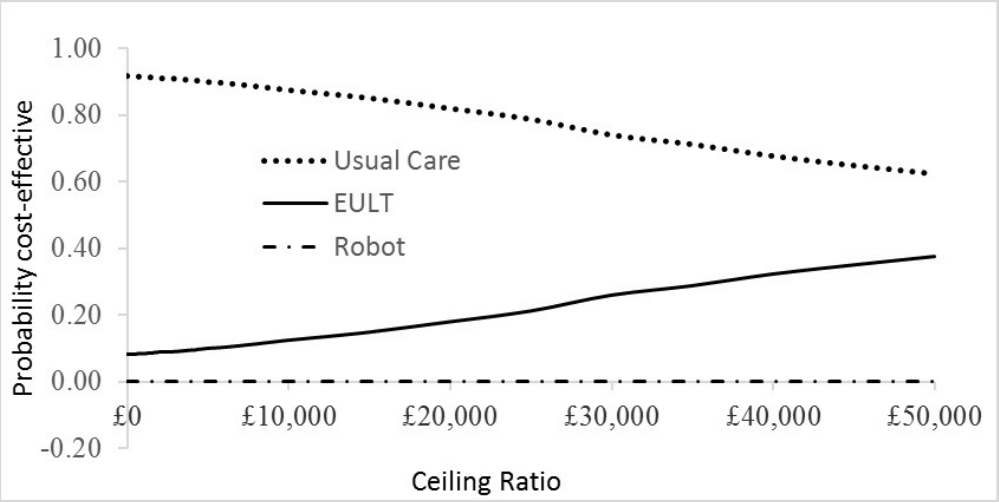

Caption : Figure 1 - Cost-effectiveness acceptability curve (base-case analysis) - adjusted bootstrapped replications for cost-effectiveness analysis

143x72mm (150 x 150 DPI)

CHEERS checklist—Items to include when reporting economic evaluations of health interventions

Section/item	Item No	Recommendation	Reported on page No/ line No
**Title and abstract**			
Title	1	Identify the study as an economic evaluation or use more specific terms such as "cost-effectiveness analysis", and describe the interventions compared.	Page 1/line 1-3
Abstract	2	Provide a structured summary of objectives, perspective, setting, methods (including study design and inputs), results (including base case and uncertainty analyses), and conclusions.	Page 2//line 23-51
**Introduction**			
Background and objectives	3	Provide an explicit statement of the broader context for the study.<br><br>Present the study question and its relevance for health policy or practice decisions.	Page 3-4/ line 56-94; Page 2 / Line 24-25
**Methods**			
Target population and subgroups	4	Describe characteristics of the base case population and subgroups analysed, including why they were chosen.	Page 2/ line 31-32; Page 6/ line 193-195
Setting and location	5	State relevant aspects of the system(s) in which the decision(s) need(s) to be made.	Page 2/ line 29, Page 13-14/ line 354-361
Study perspective	6	Describe the perspective of the study and relate this to the costs being evaluated.	Page 4/ line 98-99
Comparators	7	Describe the interventions or strategies being compared and state why they were chosen.	Page 2/ line 35-36; Page 2-3/ line 65-68
Time horizon	8	State the time horizon(s) over which costs and consequences are being evaluated and say why appropriate.	Page 2/ line 41-46; Page 4-5/ line 101-144; Page 6-7/ line 204-216
Discount rate	9	Report the choice of discount rate(s) used for costs and outcomes and say why appropriate.	Page 4 /line 109
Choice of health outcomes	10	Describe what outcomes were used as the measure(s) of benefit in the evaluation and their relevance for the type of analysis performed.	Page 2 /line 42-44; Page 5/line 135-144;
Measurement of effectiveness	11a	*Single study-based estimates:* Describe fully the design features of the single effectiveness study and why the single study was a sufficient source of clinical effectiveness data.	Page 5 /line 150-180;
	11b	*Synthesis-based estimates*: Describe fully the methods used for identification of included studies and synthesis of clinical effectiveness data.	Not applicable
Measurement and valuation of preference based outcomes	12	If applicable, describe the population and methods used to elicit preferences for outcomes.	Page 5/ line 135-144;
Estimating resources and costs	13a	*Single study-based economic evaluation:* Describe approaches used to estimate resource use associated with the alternative interventions. Describe primary or secondary research methods for valuing each resource item in terms of its unit cost. Describe any adjustments made to approximate to opportunity costs.	Page 4/ line 97-122; Page 5/all lines; Page 6/ line 166-202

Section/item	Item No	Recommendation	Reported on page No/ line No
	13b	*Model-based economic evaluation:* Describe approaches and data sources used to estimate resource use associated with model health states. Describe primary or secondary research methods for valuing each resource item in terms of its unit cost. Describe any adjustments made to approximate to opportunity costs.	Page 6-7/ 204-217
Currency, price date, and conversion	14	Report the dates of the estimated resource quantities and unit costs. Describe methods for adjusting estimated unit costs to the year of reported costs if necessary. Describe methods for converting costs into a common currency base and the exchange rate.	Page 2/ line 37; Page 5/ line 132-133
Choice of model	15	Describe and give reasons for the specific type of decision-analytical model used. Providing a figure to show model structure is strongly recommended.	Page 6 /line 204-206; Page 7/line 207-216
Assumptions	16	Describe all structural or other assumptions underpinning the decision-analytical model.	Page 6 /line 204-206; Page 7/ line 207-216
Analytical methods	17	Describe all analytical methods supporting the evaluation. This could include methods for dealing with skewed, missing, or censored data; extrapolation methods; methods for pooling data; approaches to validate or make adjustments (such as half cycle corrections) to a model; and methods for handling population heterogeneity and uncertainty.	Page 5/ line 127-129; Page 6/ line 146 - 153; Page 6/ line 171-202 Page 7/ line 5-6
**Results**			
Study parameters	18	Report the values, ranges, references, and, if used, probability distributions for all parameters. Report reasons or sources for distributions used to represent uncertainty where appropriate. Providing a table to show the input values is strongly recommended.	Information available but not included in the manuscript
Incremental costs and outcomes	19	For each intervention, report mean values for the main categories of estimated costs and outcomes of interest, as well as mean differences between the comparator groups. If applicable, report incremental cost-effectiveness ratios.	Page 9/ table 2; Page 10 /table 3; Page 11/ table 4; figure 1;
Characterising uncertainty	20a	*Single study-based economic evaluation:* Describe the effects of sampling uncertainty for the estimated incremental cost and incremental effectiveness parameters, together with the impact of methodological assumptions (such as discount rate, study perspective).	Page 10 / line 266-272; Page 11-12/ table 4; Page 12/ line 301-307; Page 13/ line 308-316
	20b	*Model-based economic evaluation:* Describe the effects on the results of uncertainty for all input parameters, and uncertainty related to the structure of the model and assumptions.	Page 11/ line 291-295; Page 11-12/ table 4;
Characterising heterogeneity	21	If applicable, report differences in costs, outcomes, or cost-effectiveness that can be explained by variations between subgroups of patients with different baseline characteristics or other observed variability in effects that are not reducible by more information.	Page 11 /line 286-295; Page 11-12/ table 4;

For peer review only

Section/item	Item No	Recommendation	Reported on page No/ line No
**Discussion**			
Study findings, limitations, generalisability, and current knowledge	22	Summarise key study findings and describe how they support the conclusions reached. Discuss limitations and the generalisability of the findings and how the findings fit with current knowledge.	Page 13/line 318-348 Page 14/ line 349-381,
**Other**			
Source of funding	23	Describe how the study was funded and the role of the funder in the identification, design, conduct, and reporting of the analysis. Describe other non-monetary sources of support.	Page 15/ line 400-403
Conflicts of interest	24	Describe any potential for conflict of interest of study contributors in accordance with journal policy. In the absence of a journal policy, we recommend authors comply with International Committee of Medical Journal Editors recommendations.	Page15/ line 411-421

For peer review only

BMJ Open

Economic evaluation of robot-assisted training versus an enhanced upper limb therapy programme or usual care for patients with moderate or severe upper limb functional limitation due to stroke: results from the RATULS randomised controlled trial

Journal:	*BMJ Open*
Manuscript ID	bmjopen-2020-042081.R1
Article Type:	Original research
Date Submitted by the Author:	02-Apr-2021
Complete List of Authors:	Fernandez-Garcia, Cristina; Newcastle University Faculty of Medical Sciences, Population Health Sciences Institute<br>Ternent, Laura; Newcastle University Faculty of Medical Sciences, Population Health Sciences Institute<br>Homer, Tara; Newcastle University Faculty of Medical Sciences, Population Health Sciences Institute<br>Rodgers, Helen; Newcastle University Faculty of Medical Sciences, Stroke Research Group - Population Health Sciences Institute; Northumbria Healthcare NHS Foundation Trust, Stroke Northumbria<br>Bosomworth, Helen; Newcastle University Faculty of Medical Sciences, Stroke Research Group, Population Health Sciences Institute<br>Shaw, Lisa; Newcastle University Faculty of Medical Sciences, Stroke Research Group, Population Health Sciences Institute<br>Aird, Lydia; Northumbria Healthcare NHS Foundation Trust, Stroke Northumbria<br>Andole, Sreeman; Barking Havering and Redbridge Hospitals NHS Trust<br>Cohen, David; London North West University Healthcare NHS Trust, Northwick Park<br>Dawson, Jesse; University of Glasgow, Institute of Cardiovascular and Medical Sciences<br>Finch, Tracy; Northumbria University, Nursing, Midwifery and Health<br>Ford, Gary; Oxford University Hospitals NHS Foundation Trust, Oxford Academic Health Science Network; Newcastle University Faculty of Medical Sciences, Stroke Research Group, Population Health Sciences<br>Francis, Richard; Newcastle University Faculty of Medical Sciences, Population Health Sciences Institute<br>Hogg, Steven ; Newcastle University Faculty of Medical Sciences, (Lay Investigator) contact Stroke Research Group, Population Health Sciences Institute<br>Hughes, Niall ; NHS Greater Glasgow and Clyde<br>Krebs, H; Massachusetts Institute of Technology,<br>Price, Christopher; Newcastle University Faculty of Medical Sciences, Stroke Research Group, Population Health Sciences; Northumbria Healthcare NHS Foundation Trust, Stroke Northumbria<br>Turner, Duncan; University of East London School of Health Sport and Bioscience<br>Van Wijck, Frederike; Glasgow Caledonian University, School of Health

	and Life Sciences<br>Wilkes, Scott; University of Sunderland, School of Pharmacy<br>Wilson, Nina; Newcastle University Faculty of Medical Sciences, Population Health Sciences Institute<br>Vale, Luke; Newcastle University Faculty of Medical Sciences, Population Health Sciences Institute

**Primary Subject Heading**:	Health economics
Secondary Subject Heading:	Cardiovascular medicine, Health services research, Rehabilitation medicine
Keywords:	STROKE MEDICINE, REHABILITATION MEDICINE, Stroke < NEUROLOGY, HEALTH ECONOMICS

SCHOLARONE™ Manuscripts

I, the Submitting Author has the right to grant and does grant on behalf of all authors of the Work (as defined in the below author licence), an exclusive licence and/or a non-exclusive licence for contributions from authors who are: i) UK Crown employees; ii) where BMJ has agreed a CC-BY licence shall apply, and/or iii) in accordance with the terms applicable for US Federal Government officers or employees acting as part of their official duties; on a worldwide, perpetual, irrevocable, royalty-free basis to BMJ Publishing Group Ltd ("BMJ") its licensees and where the relevant Journal is co-owned by BMJ to the co-owners of the Journal, to publish the Work in this journal and any other BMJ products and to exploit all rights, as set out in our licence.

The Submitting Author accepts and understands that any supply made under these terms is made by BMJ to the Submitting Author unless you are acting as an employee on behalf of your employer or a postgraduate student of an affiliated institution which is paying any applicable article publishing charge ("APC") for Open Access articles. Where the Submitting Author wishes to make the Work available on an Open Access basis (and intends to pay the relevant APC), the terms of reuse of such Open Access shall be governed by a Creative Commons licence – details of these licences and which Creative Commons licence will apply to this Work are set out in our licence referred to above.

Other than as permitted in any relevant BMJ Author's Self Archiving Policies, I confirm this Work has not been accepted for publication elsewhere, is not being considered for publication elsewhere and does not duplicate material already published. I confirm all authors consent to publication of this Work and authorise the granting of this licence.

For peer review only

Economic evaluation of robot-assisted training versus an enhanced upper limb therapy programme or usual care for patients with moderate or severe upper limb functional limitation due to stroke: results from the RATULS randomised controlled trial

Cristina Fernandez-Garcia [1], Laura Ternent[1], Tara Homer[1], Helen Rodgers[2,3,4], Helen Bosomworth[2], Lisa Shaw[2], Lydia Aird[3], Sreeman Andole[5] , David L Cohen[6], Jesse Dawson[7] ,Tracy Finch[8], Gary A Ford[2,9], Richard Francis[1], Steven Hogg[10], Niall Hughes[11], Hermano I Krebs[12], Christopher I Price[2,3], Duncan L Turner[13], Frederike van Wijck[14], Scott Wilkes[15], Nina Wilson[1], and Luke Vale[1]

[1]Population Health Sciences Institute, Newcastle University, Newcastle upon Tyne, UK, [2]Stroke Research Group, Population Health Sciences Institute, Newcastle University, Newcastle upon Tyne, UK, [3]Stroke Northumbria, Northumbria Healthcare NHS Foundation Trust, North Tyneside, UK, [4]Newcastle Hospitals NHS Foundation Trust, Newcastle upon Tyne, UK, [5]Barking, Havering and Redbridge University Hospitals NHS Trust, Romford, UK,[6]London North West Healthcare NHS Trust, Northwick Park, UK, [7]Institute of Cardiovascular and Medical Sciences, University of Glasgow, Glasgow, UK, [8]Nursing, Midwifery & Health, Northumbria University, Newcastle upon Tyne, UK, [9]Oxford Academic Health Science Network, Oxford University Hospitals NHS Foundation Trust, Oxford, UK, [10]Lay investigator (contact Stroke Research Group, Population Health Sciences Institute, Newcastle University, Newcastle upon Tyne, UK), [11]NHS Greater Glasgow and Clyde, Glasgow, UK, [12]Massachusetts Institute of Technology, Cambridge, USA, [13]School of Health, Sport and Bioscience, University of East London, London, UK, [14]School of Health and Life Sciences, Glasgow Caledonian University, Glasgow, UK, [15]School of Medicine, University of Sunderland, Sunderland, UK.

Correspondence to Dr Laura Ternent laura.ternent@newcastle.ac.uk

Word count: 4,447

For peer review only - http://bmjopen.bmj.com/site/about/guidelines.xhtml

Abstract

Objective:

To determine whether robot-assisted training is cost-effective compared with an enhanced upper limb therapy (EULT) programme or usual care.

Design:

Economic evaluation within a randomised controlled trial.

Setting:

Four National Health Service (NHS) centres in the United Kingdom (UK): Queen's Hospital, Barking, Havering and Redbridge University Hospitals NHS Trust; Northwick Park Hospital, London Northwest Healthcare NHS Trust; Queen Elizabeth University Hospital, NHS Greater Glasgow and Clyde; and North Tyneside General Hospital, Northumbria Healthcare NHS Foundation Trust.

Participants:

770 participants aged 18 or older with moderate or severe upper limb functional limitation from first-ever stroke.

Interventions:

Participants randomised to one of three programmes provided over a 12-week period: robot-assisted training plus usual care; the EULT programme plus usual care; or usual care.

Main economic outcome measures:

Mean healthcare resource use; costs to the NHS and personal social services in 2018 pounds; utility scores based on EQ-5D-5L responses; and quality-adjusted life years (QALYs). Cost-effectiveness reported as incremental cost per QALY and cost-effectiveness acceptability curves.

Results

At six months, on average usual care was the least costly option (£3,785) followed by EULT (£4,451) with robot-assisted training being the most costly (£5,387). The mean difference in total costs between the usual care and robot-assisted training groups (£1,601) was statistically significant ($p < 0.001$). Mean QALYs were highest for the EULT group (0.22) but no evidence of a difference ($p = 0.995$) was observed between the robot-assisted training (0.21) and usual care groups (0.21). The incremental cost per QALY at six months for participants randomised to EULT compared to usual care was £74,100. Cost-effectiveness acceptability curves showed that robot-assisted training was unlikely to be cost-effective and that EULT had a 19% chance of being cost-effective at the £20,000 willingness to pay (WTP) threshold. Usual care was most likely to be cost-effective at all the WTP values considered in the analysis.

Conclusions

The cost-effectiveness analysis suggested that neither robot-assisted training nor EULT, as delivered in this trial, were likely to be cost-effective at any of the cost per QALY thresholds considered.

Trial registration ISRCTN69371850

For peer review only - http://bmjopen.bmj.com/site/about/guidelines.xhtml

Strengths and limitations of this study

- Our economic evaluation was designed and conducted following best practice methods which resulted in robust and generalisable results.
- Sensitivity analyses exploring any uncertainties surrounding the level of resource use and their impact on the cost-effectiveness of the interventions add to the robustness of the results.
- The unavailability of longer term data for the within trial evaluation means that no robust inferences could be made on the long-term cost-effectiveness of the interventions.
- Poor completion of arm rehabilitation therapy logs meant that detailed information on the delivery of usual care was obtained from the health service utilisation questionnaire.
- The use of QALYs based on responses to the EQ-5D-5L questionnaire as a generic outcome measure may not accurately capture changes in quality of life for this patient group

Introduction

Stroke is the fourth leading cause of death in the UK and a leading cause of disability. Almost two thirds of stroke patients leave hospital with a disability.[1] A common disability following a stroke is loss of upper limb function. This results in a reduction of the individual's autonomy and impedes activities of daily living. Approximately 80% of people with acute stroke have upper limb motor impairment, with 50% of patients still experiencing problems after four years following the stroke.[2 3]

The Robot-Assisted Training for the Upper Limb after Stroke (RATULS) trial sought to evaluate the clinical effectiveness and cost-effectiveness of robot-assisted training (using the MIT-Manus robotic gym system) by comparing it with either an enhanced upper limb therapy (EULT) programme, or usual care.[4 5] Participants in the robot-assisted training and EULT groups also received usual care. The robot-assisted training and EULT programmes were of the same duration and frequency (45 minutes face-to-face therapy, three times a week for 12 weeks). Results from the trial provided no evidence that robot-assisted training as delivered in the study nor EULT improved upper limb function after stroke compared with usual care.[5]

After conducting a scoping review, we found little evidence of cost-effectiveness studies in the UK. The only economic evaluation we found in the literature assessed the cost-effectiveness of robot-assisted training therapy for upper limb rehabilitation within the US based VA Robotics study.[6 7] This randomised controlled trial also assessed the cost-effectiveness of the MIT-Manus robotic gym system in upper limb rehabilitation in stroke patients. Given the resource intensive nature of stroke

For peer review only - http://bmjopen.bmj.com/site/about/guidelines.xhtml

rehabilitation programmes[8] and the lifelong impacts of stroke, evidence on the cost-effectiveness of these programmes derived from well designed economic evaluations is needed. The RATULS trial is, to our knowledge, the largest and first multicentre trial with sufficient statistical power to compare robot-assisted training with another evidence based therapy programme, or usual care.[9] This paper reports the results from a within-trial analysis that formed part of the RATULS trial.

Trial overview

Summary of RATULS Trial

The study was a three arm randomised controlled trial (RCT) which recruited 770 participants from stroke units, day hospitals, outpatient clinics, primary care, community rehabilitation services and local stroke clubs in four study centres. The sample size calculation yielded a target sample size of 762 participants with 216 participants in each group required to provide 80% power at a significance level of 1.7%. The sample size was revised after protocol publication to 770 to allow for 15% attrition (rather than 10% as originally specified in the published protocol). Full details on sample size calculation and trial methodology have been reported elsewhere.[4 5]

Participants were eligible for inclusion in the study if they were 18 years or older, had experienced a first-ever stroke between one week and five years prior to randomisation and, as a consequence of the stroke, had moderate or severe upper limb functional limitation as measured by the Action Research Arm Test (ARAT)[10] (score 0-39). Potential participants who had been previously enrolled in this study; had participated or were participating in another upper limb rehabilitation study; had previously used the MIT-Manus robotic gym or another arm rehabilitation robot; had other notable upper limb impairment; or had a diagnosis that would interfere with the rehabilitation or outcome assessments were excluded from the trial. All those who were eligible and who consented to participate in the study were randomised on a 1:1:1 ratio to receive one of the three interventions.

The clinical primary outcome of the study was upper limb function "success" using (ARAT)[10] at three months post randomisation. The definition of success differed depending on baseline severity of upper limb functional limitation. Success for a baseline ARAT score 0–7 was defined as an improvement of 3 points or more; a baseline ARAT 8–13 required an improvement of 4 points or more; baseline ARAT 14–19 required an improvement of 5 points or more; and finally a baseline ARAT 20–39 required an improvement of 6 points or more.[4 5] Full details of the clinical trial results and methodology have been described elsewhere.[4 5]

Economic evaluation methods

We conducted an economic evaluation consisting of a cost-utility analysis using the QALY as the primary outcome measure following guidance for best practice in health technology appraisal.[11]

Perspective:

We conducted all analyses adopting the perspective of the UK National Health Service (NHS) and Personal Social Services (PSS) setting.

Costs

The costs included in the analysis comprised the intervention costs for the robot-assisted training and EULT programmes, no intervention costs were directly associated with usual care and we assumed that any rehabilitation received had been reported in the health service utilisation

For peer review only - http://bmjopen.bmj.com/site/about/guidelines.xhtml

questionnaire. We also included use of health and social services over the six month follow-up period for all randomised groups. For the intervention costs we assumed, as per the protocol,[4] that each therapy session in both intervention groups lasted 60 minutes including 45 minutes of face-to-face therapy on a 1:1 basis including the same staff component. In addition to staff time, capital costs were calculated for robot-assisted training using the "equivalent annual cost"[12] methodology applying a 3.5% discount rate and on the basis that the equipment would need replacing after five years.

We developed a health service utilisation questionnaire, informed by previous data collection tools including the Client Service Receipt Inventory (CSRI)[13] to capture health and social care resource use at baseline and six months post-randomisation. At baseline, participants were asked about the care received in the three months before they joined the study. These data were used to control for any imbalance in participant use of services at baseline. The information collected at six months included: visits to Accident & Emergency (A&E) departments; outpatient appointments; day patient appointments; overnight hospital stays; the use of GP and nursing services; use of therapy services; medications; community based healthcare; social services; and residential care and nursing home stays.

To estimate the cost at six months for each participant we combined data on use of care and services with unit costs obtained from routine data sources. We applied NHS national reference costs[14] to hospital based services. Information for staff costs, unit costs relating to primary care, social care, and community health services was mostly derived from Curtis et.al. 2017.[15] In order to cost prescribed medication, we collected information on medication name, dosage, frequency and intake duration. We combined this information with unit costs taken from the British National Formulary.[16] When information on intake duration, medication format (e.g. dose; mode of intake) was missing, we used prescription cost analysis data[17] and assumed that the participant had been taking the most commonly prescribed format and had been issued one prescription for the trial period.

We used the information collected at six month follow-up in addition to the intervention costs to derive the total mean cost per participant per each randomised group. All costs are reported in pounds Sterling and converted to 2018 prices, when appropriate, using the Bank of England inflation calculator.[18]

Effectiveness outcome

The quality of life outcome measures used for the economic evaluation were the summary utility scores derived from the EQ-5D-5L questionnaires.[19] These were completed by participants at baseline, three and six months post-randomisation. The EQ-5D-5L questionnaire measures the individual's self-reported health-related quality of life through the following five domains: mobility, self-care, usual activities, pain/discomfort, and anxiety/depression. Respondents were asked to describe each domain according to five different levels: no problems, slight, moderate, severe, or extreme problems. Since there is no currently accepted valuation set for the EQ-5D-5L questionnaire, we mapped the questionnaire responses to the E5-5D-3L[20 21] descriptive system in order to generate the utility values. These utility values formed the basis of our QALY calculations using the area-under-the-curve approach.[22]

Missing data

For the main (base-case) cost-effectiveness analysis we only included those participants for whom we had some data on costs. Once we determined that missing cost data were missing at random, we

For peer review only - http://bmjopen.bmj.com/site/about/guidelines.xhtml

explored in the sensitivity analyses the effect on cost-effectiveness of applying multiple imputation to missing total costs controlling for age, sex and baseline ARAT score.

We explored the patterns of missing utility data. Once we established that information was missing at random, we used multiple imputation methods to estimate the missing utility values at three months and six months. This involved applying truncated normal regression whilst controlling for age, sex, and baseline ARAT score in order to generate the missing utility value.

Base-case cost-effectiveness analysis

We calculated mean costs and effects along with corresponding standard deviations. Where we report differences in mean costs and effects between all three randomised groups we used 98.33% confidence intervals, as this was a three arm comparison. We conducted all pairwise comparisons using 95% confidence intervals. Using seemingly unrelated regression modelling methodology[23] in the adjusted cost-effectiveness analysis, we derived the incremental cost per gained QALY for each participant at six months. This approach involved estimating two linear regressions with their own dependent variable for costs and QALYs and a set of explanatory variables. We used randomised group, study centre, and time since stroke as explanatory variables for both costs and QALYs. Additionally, we incorporated baseline utility scores as an explanatory variable for the QALY equation and total baseline costs as an explanatory variable to the costs equation. We presented the results from the adjusted analysis in the form of an incremental cost-effectiveness ratio (ICER). The ICER is the difference in mean costs divided by the difference in mean effects (in this case QALYs) between two alternatives.

In the analysis if a comparator was both more costly and less effective than the others it was dropped from any further cost-effectiveness comparisons because it was less cost-effective than the other comparator.

We created cost-effectiveness acceptability curves in order to assess the imprecision surrounding the estimates of costs, effects and cost-effectiveness. This approach involved drawing bootstrapped samples, with replacement, of the mean costs and mean QALYs from the original trial data. We repeated this process increasing the number of replications until the results were stable. This was achieved at 1,000 replications. After using the new values generated from the bootstrapping exercise to calculate the difference in costs and effects between groups, we combined this information with a range of willingness to pay (WTP) values (£0, £10,000, £20,000, £30,000, £50,000) per QALY gained. This involved using the net benefit statistic[24] which multiplied the gain in health (QALYs) by the chosen WTP value, the incremental cost was then subtracted to obtain the net monetary benefit. We used these results to generate a cost-effectiveness acceptability curve which graphically represented the probability of each of the interventions being cost-effective at each of the pre-specified value for society's WTP for a QALY.[25] All analyses were carried out in Stata 15.[26]

Sensitivity, subgroup and per-protocol analyses

We conducted deterministic sensitivity in order to assess the robustness of the cost-effectiveness results for three scenarios.

First, we examined the impact of assigning a value of zero to missing total healthcare costs.

Second, we examined the possibility that those participants with missing total healthcare costs may have used some services and hence incurred some costs. Under this scenario, once we established that information was missing at random, we applied truncated normal regression methods excluding

For peer review only - http://bmjopen.bmj.com/site/about/guidelines.xhtml

total costs values below zero and above £25,000 (as the highest observed value for total costs) and used age, sex and baseline ARAT as covariates.

Finally, we investigated whether increasing the life span of the MIT-Manus robotic gym system from five to seven years would affect the cost-effectiveness of the interventions.

In order to explore the impact of time since stroke on the cost-effectiveness of the interventions, we conducted an exploratory analysis for the subgroups outlined in the protocol.[4] Three subgroups were pre-specified (< 3 months, 3-12 months and >12 months) according to time since stroke.

A secondary per-protocol cost-effectiveness analysis removing from the data set those participants who did not receive at least 20 sessions of therapy in the robot-assisted training and the EULT programme groups was also conducted. The cut-off point of 20 sessions was based on clinical evidence that an additional 20 hours of therapy compared with control interventions leads to improvements on functional outcome.[27]

We combined each sensitivity, subgroup and per-protocol analyses with bootstrapping in order to reflect the imprecision surrounding the cost-effectiveness results.

Extrapolation of trial results

We carried out a modelling exercise designed to extrapolate the mean QALYs at six months to 12 months based on the results of the trial, for this, we made a number of assumptions. First, we assumed that participants across all groups maintained the same utility levels reported at six months post-randomisation. Second, we considered all intervention costs and therapy related costs (physiotherapy, occupational therapy, and speech and language therapy) as sunk costs, since they were deemed not to continue beyond the six month trial period. However, we assumed that all other levels of healthcare resource use remained constant from six to 12 months post randomisation.

We calculated the difference in mean costs and effects between the randomised groups with differences across groups not being formally tested. We used seemingly unrelated regression[23] methods to calculate the adjusted ICER at 12 months including the same explanatory variables used in the base-case cost-effectiveness analysis. As in the base-case analysis we used the bootstrapping technique to present uncertainty surrounding mean costs, effects, and cost-effectiveness.

Patient and Public Involvement

We have designed and reported our research with input from stroke survivors. Members from the North East Stroke Research Network Patient and Carer Panel provided input to the design and content of trial documents, including health economics questionnaires.

Results

Completeness of data

Most participants (96%) across all groups completed the health service utilisation questionnaire at baseline. There was a progressive increase in non-responses to the health service utilisation questionnaires over the six month follow up period. The pattern of responses was similar across the intervention groups however, the loss to follow-up was more pronounced in the usual care group. Whilst completion rates were 83% for the robot-assisted training group and 85% for the EULT group, this decreased to 70% of usual care participants. Completion rate of EQ-5D-5L at baseline was 99%

For peer review only - http://bmjopen.bmj.com/site/about/guidelines.xhtml

across all of participants. This decreased to 88% at three months and 82% at six months. The highest number of non-responders belonged to the usual care group with response rates of 81% at three months and 75% at six months.

Health service use and costs.

Reported health service resource use was broadly similar across all randomised groups at six months (Table 1). Large standard deviations indicate that there was substantial variation in use of service between individuals in all three randomised groups, with a few participants reporting very high use of some services. Whilst not statistically tested, the main apparent difference between groups was in the therapy services received, with usual care participants receiving more home physiotherapy and speech and language therapy sessions compared with the robotic-assisted training and EULT groups. Participants in usual care had a higher reported mean number of contacts with general practice and nursing services compared with the robotic-assisted training and EULT groups.

Table 1- Reported health service resource use at six months

**Area of resource utilisation**	**Robot-assisted training (n=257)**		**EULT (n=259)**		**Usual care (n=254)**	
	Respondents n*	Mean contacts (SD)	Respondents n*	Mean contacts (SD)	Respondents n*	Mean contacts (SD)
GP surgery	205	1.80 (3.88)	208	1.49 (2.08)	168	1.83 (2.17)
GP home	209	0.37 (1.08)	213	0.27 (0.73)	174	0.38 (1.29)
GP phone	207	0.53 (1.22)	207	0.39 (0.96)	172	0.30 (0.77)
Nurse surgery	203	0.61 (1.39)	210	0.49 (1.19)	169	0.90 (5.58)
Nurse home	206	0.47 (2.07)	213	0.45 (1.65)	170	5.39 (44.63)
Nurse phone	211	0.08 (0.56)	212	0.52 (0.31)	174	0.04 (0.25)
NHS direct	211	0.11 (0.44)	213	0.08 (0.35)	174	0.03 (0.32)
Physiotherapy hospital	210	2.18 (6.10)	211	2.99 (9.23)	171	2.64 (7.72)
Physiotherapy home	205	2.76 (8.76)	207	3.10 (8.04)	170	4.35 (11.87)
Physiotherapy at general practice surgery	213	0.13 (1.16)	212	0.40 (2.63)	176	0.50 (3.85)
Physiotherapy elsewhere	212	0.23 (1.45)	213	0.21 (1.82)	175	0.62 (4.63)
Occupational therapy hospital	211	0.64 (3.14)	211	1.23 (7.23)	174	0.57 (3.53)
Occupational therapy home	210	1.56 (5.39)	209	1.27 (4.28)	173	1.95 (7.23)
Occupational therapy at general practice surgery	213	0.00 (0.07)	211	0.01 (0.14)	176	0.02 (0.23)
Occupational therapy elsewhere	212	0.16 (1.49)	211	0.00 (0.00)	176	0.00 (0.00)
Speech and language therapy hospital	213	0.57 (2.79)	213	0.65 (3.74)	173	0.94 (5.24)
Speech and language therapy home	210	0.47 (2.00)	212	0.64 (3.97)	175	2.22 (15.42)
Speech and language therapy at general practice surgery	212	0.00 (0.00)	213	0.03 (0.42)	176	0.02 (0.13)

For peer review only - http://bmjopen.bmj.com/site/about/guidelines.xhtml

Speech and language therapy elsewhere	213	0.05 (0.42)	213	0.47 (0.68)	175	0.03 (0.45)
A&E visits	213	0.33 (0.77)	214	0.37 (0.98)	178	0.24 (0.70)
Outpatient appointments	212	1.64 (3.14)	215	1.42 (2.88)	176	1.48 (4.24)
Hospital nights after being admitted via A&E	213	0.79 (4.77)	215	1.83 (12.95)	176	0.70 (3.59)
Hospital nights NOT admitted via A&E	213	0.28 (3.09)	215	0.03 (0.19)	176	0.25 (1.81)
Day patient treatment (half day)	205	0.08 (0.35)	210	0.09 (0.46)	175	0.06 (0.32)
Day patient treatment (full day)	199	0.03 (0.17)	201	0.02 (0.18)	169	0.06 (0.07)
Residential care	213	1.75 (14.65)	215	2.08 (17.07)	177	1.39 (10.72)
Nursing home	213	0.00 (0.00)	216	0.83 (12.24)	177	3.08 (23.51)
Meals on wheels	213	0.02 (0.27)	213	0.04 (0.49)	177	0.08 (1.05)
Home help personal care	211	2.89 (6.77)	210	3.04 (7.42)	173	3.17 (7.03)
Home help household tasks	213	0.74 (3.21)	212	0.68 (3.87)	175	1.06 (4.54)
Home help shopping	213	0.11 (0.74)	212	0.07 (0.53)	176	0.11 (0.76)
Health visitor	212	0.05 (0.45)	213	0.09 (0.85)	177	0.03 (0.28)
Geriatrician	212	0.03 (0.42)	213	0.02 (0.23)	177	0.00 (0.00)
Psychiatrist	212	0.13 (0.72)	213	0.75 (0.54)	175	0.01 (0.11)
Psychologist	209	0.45 (2.07)	211	0.28 (1.14)	176	0.44 (2.37)
Chiropodist	210	0.55 (1.19)	210	0.39 (1.06)	173	0.44 (1.02)
Optician	210	0.25 (0.56)	212	0.23 (0.53)	174	0.32 (0.64)
Pharmacist	211	0.66 (2.33)	207	0.60 (2.28)	174	1.16 (4.35)

n*= Number of participants who completed all or part of the questionnaire
SD: Standard deviation; GP: General practitioner; A&E: Accident and Emergency

The mean total cost per participant for each randomised group is reported in Table 2. We also report the mean total cost per cost category per participant. The highest mean costs per participant were associated with the use of social care services which included stays in residential and nursing home facilities and care assistance received at home. The average cost per participant was higher in the usual care group in all categories except in secondary care and other NHS and social services used by the participants. The addition of the intervention costs however, reversed this finding making the mean cost per participant highest in the group receiving robot-assisted training. We found some evidence the mean difference in costs between the robot-assisted training group and the usual care group was higher (Mean difference: 1,601 [95% CI 706 to 2,496]). However, there was no evidence of a difference between the EULT group and usual care higher (Mean difference: 665 [95% CI -444 to 1,774]).

Table 2 – Total cost (£) over six months for all participants with full economic data

Area of resource utilisation	Robot-assisted training (n= 257)		EULT (n=259)		Usual care (n=254)	
	n	Mean (SD)	n	Mean (SD)	n	Mean (SD)
Intervention costs	257	2,872	259	1,399	0	-(-)

For peer review only

For peer review only - http://bmjopen.bmj.com/site/about/guidelines.xhtml

		(0)		(0)		
Primary care costs and community based health care (including therapy services)	213	743 (1,031)	215	777 (1,262)	177	1,078 (1,813)
Social care	213	1,410 (3,146)	216	1,541 (3,943)	178	1,890 (4,281)
Secondary care	213	733 (2,247)	216	988 (4,486)	178	668 (1,880)
Medication costs	157	149 (302)	162	154 (273)	126	198 (347)
Other NHS and social services	11	727 (983)	13	790 (946)	9	307 (406)
Deceased participants	1	0 (0)	3	13,953 (4,516)	0	- (-)
Mean total cost	257	5,387 (4,054)	259	4,451 (6,033)	178	3,785 (5,437)
Mean difference between robot-assisted training and usual care with 95% confidence interval; p-value	1,601 [706 to 2,496]; < 0.001					
Mean difference between EULT and usual care with 95% confidence interval; p-value	665 [-444 to 1,774]; 0.239					
SD: Standard deviation; n= randomised n						

Health outcomes.

The mean utility scores across all randomised groups were similar at baseline, three months and six months as were mean QALYs (Table 3). The EULT group had the highest mean QALY (0.23) followed by robot-assisted training and usual care groups, both of which had a mean QALY over six months of 0.21. The mean differences in QALYs between each of the intervention groups (robot-assisted training and EULT) and usual care were found to be very small and there was no evidence of a difference between randomised groups.

Table 3 - Utility scores at baseline, three months and six months and QALYs at six months

Time period	Robot-assisted training (n*=257)		EULT (n*=259)		Usual care (n*=254)	
	n	Mean (SD)	n	Mean (SD)	n	Mean (SD)
Baseline EQ-5D-5L score	254	0.36 (0.26)	259	0.39 (0.25)	254	0.37 (0.26)
3 month EQ-5D-5L score	232	0.45 (0.27)	236	0.48 (0.24)	207	0.42 (0.29)
6 month EQ-5D-5L score	223	0.46 (0.29)	222	0.50 (0.27)	190	0.46 (0.27)
QALYs at 6 months after multiple imputation	254	0.21 (0.12)	259	0.23 (0.10)	254	0.21 (0.11)
Mean difference in QALYs between robot-assisted training and usual care with 95% confidence interval; p-value	0.00 (-0.20 to 0.20); 0.995					

For peer review only - http://bmjopen.bmj.com/site/about/guidelines.xhtml

Mean difference in QALYs between EULT and usual care with 95% confidence interval; p-value	0.02 (0.00 to 0.35); 0.080
n*= number randomised; SD: Standard deviation; QALY: Quality adjusted life year	

Cost-effectiveness analysis, subgroup analysis, per-protocol analysis and longer term model.

The base-case cost-effectiveness analysis results (Table 4) show the adjusted ICER was £74,100 for the comparison between EULT and usual care. Robot-assisted therapy was, on average, dominated by EULT since it was both, on average, more costly and less effective. The cost-effectiveness acceptability curve (Figure 1), shows that EULT had a 19% chance of being cost-effective at the £20,000 WTP threshold value. The probability of EULT being cost-effective remained below 40% at all the WTP values considered in the analysis. Robot-assisted training had no probability of being cost-effective at all WTP values considered in the analysis.

The results from the subgroup analysis are also summarised in Table 4. These showed that robot-assisted training remained dominated on average by EULT in both the adjusted and unadjusted results for all subgroups. The highest ICER (£126,143) comparing EULT with usual care was linked to those participants who had a stroke more than 12 months before randomisation. The subgroup of participants who were less than three months post-stroke at randomisation had the lowest ICER (£31,400) for the comparison of EULT with usual care. The bootstrapped sensitivity analysis for this group suggested that EULT had a 41% probability of being cost-effective at the £20,000 WTP threshold.

Results from the per-protocol analysis did not change the direction of the cost-effectiveness results. Usual care remained the least costly option followed by EULT and robot-assisted training. The ICER for EULT and usual care was £68,000 and EULT only had a 17% probability of being cost effective at the £20,000 WTP threshold. The probability of robot-assisted therapy being considered cost-effective was very low.

Table 4 shows the results from the economic model which extrapolated the trial data on costs and effects to 12 months. Unadjusted mean costs per participant were lowest in the EULT group (£6,892) followed closely by usual care (£6,916) and robot-assisted training (£7,538). Mean QALYs were in line with those seen in the base-case analysis with participants in the EULT group having highest mean QALYs at 12-months (0.48) followed by usual care (0.47) and robot-assisted training (0.44). Once we adjusted for baseline costs, baseline utility score, study centre, randomised group and time since stroke, usual care reverted to being the least costly option. The ICER for the comparison between EULT and usual care was £6,095, however there was only a 55% probability of EULT being considered cost-effective compared with usual care at the £20,000 WTP value. Robot-assisted training had no probability of being cost-effective at this WTP value.

Table 4 - Results from base-case cost-effectiveness analysis, subgroup analyses and longer term economic model

Scenario	Robot-assisted training	EULT (n=259)	Usual care (n=254)	ICER	Probability of each therapy being cost-effective at the £20,000 WTP threshold

For peer review only - http://bmjopen.bmj.com/site/about/guidelines.xhtml

(n=257)					Robot-assisted training	EULT	Usual care
Base-case analysis							
Cost – £, unadjusted, mean (CI)*	5,387 (4,777 to 5,996)	4,451 (3,548 to 5,354)	5,387 (4,777 to 5,996)	-	-	-	-
QALY - unadjusted, mean (CI)*	0.21 (0.195 to 0.229)	0.23 (0.213 to 0.244)	0.21 (0.194 to 0.230)	-	-	-	-
ICER (£ per QALY) – adjusted EULT vs Usual care§	-	-	-	74,100	0.00	0.19	0.81
Subgroup analysis (Less than 3 months - time since stroke)							
Cost – £, unadjusted, mean (CI)	5,485 (3,938 to 7,032)	3,863 (2,527 to 5,199)	3,328 (1,443 to 5,213)	-	-	-	-
QALY - unadjusted, mean (CI)*	0.22 (0.19 to 0.25)	0.24 (0.20 to 0.28)	0.21 (0.17 to 0.25)	-	-	-	-
ICER (£ per QALY) – adjusted EULT vs Usual care§	-	-	-	31,400	0.00	0.41	0.59
Subgroup analysis (3 to 12 months - time since stroke)							
Cost – £, unadjusted, mean (CI)*	5,790 (4,793 to 6,786)	5,084 (3,393 to 6,774)	4,943 (3,228 to 6,658)	-	-	-	-
QALY - unadjusted, mean (CI)	0.20 (0.18 to 0.23)	0.23 (0.20 to 0.25)	0.21 (0.19 to 0.24)	-	-	-	-
ICER (£ per QALY) – adjusted EULT vs Usual care§	-	-	-	79,400	0.04	0.37	0.59
Subgroup analysis (More than 12 months)							
Cost – £, unadjusted, mean (CI)*	4,822 (4,036 to 5,728)	3,961 (2,783 to 5,138)	2,823 (1,299 to 4,348)	-	-	-	-
QALY - unadjusted, mean (CI)*	0.22 [0.18 to 0.25]	0.23 [0.20 to 0.25]	0.21 (0.18 to 0.24]	-	-	-	-
ICER (£ per QALY) – adjusted EULT vs Usual care§	-	-	-	126,143	0.01	0.15	0.84
Per-protocol analysis							
Cost – £, unadjusted, mean (CI)*	5,595 (4,929 to 6,261)	4,551 (3,596 to 5,501)	3,785 (2801 to 4770)	-	-	-	-
QALY - unadjusted, mean (CI)*	0.22 (0.20 to 0.24)	0.23 (0.21 to 0.25)	0.21 (0.19 to 0.23)	-	-	-	-
ICER (£ per QALY) – adjusted EULT vs UC§	-	-	-	68,000	0.00	0.17	0.83
Extrapolation to 12 months							

For peer review only - http://bmjopen.bmj.com/site/about/guidelines.xhtml

Cost – £, unadjusted, mean (CI)*	7,538 (6,350 to 8,725)	6,892 (5,149 to 8,635)	6,916 (5,003 to 8,830)	-	-	-	-
QALY - unadjusted, mean (CI)*	0.44 (0.40 to 0.48)	0.48 (0.44 to 0.51)	0.45 (0.41 to 0.48)	-	-	-	-
ICER (£ per QALY) – adjusted EULT vs Usual care§	-	-	-	6,095	0.10	0.55	0.35

QALY: quality adjusted life year
CI: confidence interval
ICER: Incremental cost-effectiveness ratio
WTP: Willingness to pay
* 98.33% confident interval used throughout the analyses for the three arm comparison
§ data adjusted for baseline costs, baseline utility score, study centre, randomised group and time since stroke

(Insert Figure 1)

Sensitivity analyses

The different scenarios explored in these analyses did not change the direction of the results from the base-case cost-effectiveness results. Robot-assisted training remained dominated on average by EULT in all instances. First, when we changed the missing costs to zero, the resulting ICER between EULT and usual care increased to £172,000. This increase is to be expected since all participants with missing total costs belong to the usual care group. By imputing zero for missing costs the mean costs for the usual care participants decreased and hence, when this was done, the ICER increased when compared to EULT.

Second, applying multiple imputation methods to missing costs resulted in an increase to the unadjusted mean usual care costs (£4,451) compared with the base-case results (£3,785). Consequently, the resulting ICER from the comparison between EULT and usual care decreased to £50,000 with the probability of EULT being cost-effective at £20,000 increasing to 27%.

Third, extending the life of the robotic gym system resulted in a reduction of the mean capital costs per patient and hence, in a lower mean total cost for the robot-assisted training group (£5,085) compared with the base-case analysis (£5,387). There were no changes to the utility scores across all groups nor to the mean costs for EULT and usual care, hence the resulting ICER for the comparison of EULT and usual care remained the same as in the base-case analysis (£74,100).

Discussion

The RATULS trial found no evidence that robot-assisted training, as delivered in the study, improved upper limb function “success” for stroke patients with moderate or severe upper limb functional limitation when compared with a EULT programme or usual care.[5] Our economic evaluation strengthens the evidence base of these upper limb rehabilitation programmes through the evaluation of their cost-effectiveness.

For peer review only - http://bmjopen.bmj.com/site/about/guidelines.xhtml

Results from the base-case cost-effectiveness analysis suggested that, on average, robot-assisted training was more costly than both EULT and usual care and that robot-assisted training was slightly less effective than EULT. EULT was on average more costly and as effective as usual care in the unadjusted analyses and more costly and more effective in the adjusted analyses. The balance of probabilities favoured usual care as the preferred upper limb rehabilitation therapy over the range of WTP values considered. Focusing on the society's WTP for a QALY, the bootstrapped analysis suggested that, EULT, as delivered in this trial, was unlikely to be cost-effective over any of the WTP values considered, despite being more effective than robot-assisted training and usual care. The subgroup, sensitivity and per-protocol analyses did not change the direction of the base-case cost-effectiveness results. Extrapolating within-trial results to 12 months produced the lowest ICER for the comparison between EULT and usual care overall, however, high uncertainty surrounds the assumptions made about how costs and utilities change beyond the trial follow-up.

The main strength of this economic evaluation is that it was conducted as part of a rigorously run RCT and followed guidelines for best practice throughout.[11 28] As a result, we were able to base the economic evaluation on individual patient data collected during the trial and benefited from low levels of missing healthcare resource use and quality of life data. However, the loss to follow up in the usual care group may have led to an underestimation of resource use for these participants. Nevertheless, when we imputed missing values the results were still consistent with those from the base-case analysis.

One of the main challenges in conducting the economic evaluation was the difficulty to ascertain the specific components of usual care therapy. Log books designed to capture detailed usual care information were completed by participants. The information gathered was to be used alongside the health service utilisation questionnaire. Completion rates, however, were very low and we were unable to incorporate these data into the economic evaluation. We overcame this by drawing on the information captured via our primary data collection tool, the healthcare service utilisation questionnaire, where participants recorded any therapy sessions received during the trial period.

Through the collection of self-reported quality of life information at three points during the study using the EQ-5D-5L questionnaire,[19] we were able to measure quality of life gains for participants across all groups. One strength of this generic tool is that decision makers will be able to make priority-setting decisions not only for this patient group but across different disease areas.[11] However, the EQ-5D-5L questionnaire does not capture transitory changes as it only asks about health on the day the participant completes it. In addition, this questionnaire and the QALYs derived from it are not stroke specific and it is unknown whether we were able to accurately capture changes in quality of life in this patient group.

A noteworthy limitation of the economic evaluation is associated with the timeframe of the trial. The within-trial economic evaluation assessed the cost-effectiveness of the interventions at six months. A longer term perspective was originally planned but due to limitations of the data, extrapolation to 12 months only was conducted. The results however, need to be interpreted with caution due to the assumptions made on both costs and utility values.

The economic evaluation fills a significant evidence gap with this being the first economic evaluation comparing robot-assisted training with usual care having been conducted in the UK NHS setting. This evaluation expands the analysis conducted on the cost-effectiveness of the MIT-Manus robotic system as part of the VA Robotics trial.[6 7] This study, confined to the US healthcare system, reported a small QALY gain for the robot group compared to usual care and not significant differences in costs between groups. Differences in the healthcare system between both countries means that our

For peer review only - http://bmjopen.bmj.com/site/about/guidelines.xhtml

economic evaluation is key for making cost-effectiveness results relevant to the UK NHS setting. Furthermore, it takes into account a number of key differences in the design of both studies. First, whilst the VA robotics trial assumed that the robotic gym could be used simultaneously by two patients, the RATULS trial was designed to deliver robot-assisted training on a one-to-one basis. Second, the components of the intensive comparison therapy differed in each study. Third, we used E5-5D-5L as the recommended tool to calculate QALYs, whilst the VA robotic trial calculated QALYs from responses to the Health Utility Index Mark 3 (HUI3) questionnaire.[29] All these points reduce the grounds for comparability between studies and supports the need for the economic analysis we conducted alongside the RATULS trial.

The use of multiple sites contributed to the generalisability of the economic evaluation. The analyses controlled for differences in sites hence minimising the chance of obtaining biased results from differences in costs and effects driven by location.

In conclusion, robot-assisted training was not found to be cost-effective in comparison to EULT and usual care. This economic evaluation suggested that usual care remained the most cost-effective type of upper limb rehabilitation compared with a EULT therapy programme and robot-assisted training for stroke patients with moderate or severe functional limitation.

The results create opportunities for further research. In particular, further research could explore the potential effect on both costs and QALYs from reconfigurations to the delivery of EULT and robot-assisted training. It remains unclear, for example, whether delivering therapy in a group setting may first, be feasible in the NHS setting and second improve the quality of life and clinical outcomes. Further development of these interventions may increase their cost-effectiveness compared to usual care. Additionally, studies with a longer follow-up data may help establish whether the QALY gains derived from the interventions are sustained beyond the set timeframe of the trial, this can then lead to a robust assessment of their long-term cost-effectiveness.

Figure 1 - Cost-effectiveness acceptability curve (base-case analysis) - adjusted bootstrapped replications for cost-effectiveness analysis

For peer review only - http://bmjopen.bmj.com/site/about/guidelines.xhtml

Contributors:

CFG conducted the health economic evaluation. LV and LT are senior health economists and were involved in the main study design, delivery, analysis and interpretation of the data and drafting the manuscript. TH contributed to the health economic evaluation and drafting of the manuscript. HR was the Chief Investigator of the study. HB, HIK, FvW , GAF, LS, LA, TF, CIP, DLT, SW, JD, were involved in the main study design, delivery, data management, data analysis and drafting the manuscript. NW, SA, DLC, RF, NH contributed to study delivery, interpretation of the data and drafting the manuscript. SH was a co-investigator and is a service user, he was involved in the main study design and delivery, interpretation of data. All authors have commented upon drafts of the manuscript and have given final approval to this version.

Acknowledgements:

We would like to thank Jenni Hislop for her input into the health economics analysis plan and the development of the data collection tools.

Funding statement:

This work was supported by the National Institute for Health Research Health Technology Assessment Programme (reference: 11/26/05). The views and opinions expressed here are those of the authors and do not necessarily reflect those of the HTA programme, NIHR, the UK National Health Service (NHS), or UK Department of Health.

Data availability statement:

De-identified participant data will be made available to scientific researchers upon approval of their study protocol and analysis plan, by a committee of the RATULS team. Proposals should be directed to the corresponding author. A data sharing agreement will need to be signed by data requestors. Requests for data sharing will be considered after publication of the full trial report in the Health Technology Assessment Journal which is anticipated in 2020.

Competing interests:

Professor Rodgers reports grants from NIHR, during the conduct of the study; personal fees from Bayer, outside the submitted work; and Member of NIHR HTA CET panel 2010 to 2014. Professor Ford reports grants from National Institute for Health Research, during the conduct of the study; personal fees from Amgen, personal fees from Daiichi Sankyo, grants and personal fees from Medtronic, personal fees from Stryker, personal fees from Pfizer, personal fees from Bayer, outside the submitted work. Professor Price and Dr Lisa Shaw report grants from National Institute for Health Research, during the conduct of the study. Professor Krebs reports other from Interactive Motion Technologies, other from 4Motion Robotics, outside the submitted work; in addition, Professor Krebs has a patent Interactive Robotic Therapist; US Patent 5,466,213; 1995; Massachusetts Institute of Technology issued, and a patent Wrist And Upper Extremity Motion; US Patent No. 7,618,381; 2009; Massachusetts Institute of Technology licensed to Bionik Laboratories.

Ethics Statement:

Ethical approval was obtained for the clinical trial (Trial registration ISRCTN69371850) and the economic questionnaires. Approval was granted by the National Research Ethics Committee Sunderland (reference 12/NE/0274).
The research here submitted is confined to the within trial economic evaluation.

For peer review only - http://bmjopen.bmj.com/site/about/guidelines.xhtml

References

1. Stroke Association, UK. State of the nation stroke statistics - February 2018. Available from: https://www.stroke.org.uk/system/files/sotn_2018.pdf. Accessed 15th September 2018.

2. Langhorne P, Coupar F, Pollock A. Motor recovery after stroke: a systematic review. *The Lancet Neurology* 2009;8:741-54.

3. Broeks J, Lankhorst G, Rumping K, et al. The long-term outcome of arm function after stroke: results of a follow-up study. *Disability and Rehabilitation* 1999;21(8):357-64.

4. Rodgers H, Shaw L, Bosomworth H, et al. Robot assisted training for the upper limb after stroke (RATULS): study protocol for a randomised controlled trial. *Trials* 2017;18:340.

5. Rodgers H, Bosomworth H, Krebs HI, et al. Robot assisted training for the upper limb after stroke (RATULS): a multicentre randomised controlled trial. *The Lancet* 2019;394:51-62.

6. Wagner TH, Lo AC, Peduzzi P, et al. An economic analysis of robot-assisted therapy for long-term upper-limb impairment after stroke. *Stroke* 2011;42(9):2630-32.

7. Lo AC, Guarino P, Krebs HI, et al. Multicenter randomized trial of robot-assisted rehabilitation for chronic stroke: methods and entry characteristics for VA ROBOTICS. *Neurorehabilitation and Neural Repair* 2009;23(8):775-83.

8. Craig LE, Wu O, Bernhardt J, et al. Approaches to economic evaluations of stroke rehabilitation. *International Journal of Stroke* 2014;9(1):88-100.

9. Rodgers H, Bosomworth H, Krebs HI, et al. Robot-assisted training compared with an enhanced upper limb therapy programme and with usual care for upper limb functional limitation after stroke: the RATULS three-group RCT. *Health technology assessment (Winchester, England)* 2020;24(54):1.

10. Lyle RC. A performance test for assessment of upper limb function in physical rehabilitation treatment and research. *International Journal of Rehabilitation Research* 1981;4(4):483-92.

11. NICE. Guide to the methods of technology appraisal 2013: National Institute for Health and Care Excellence, 2013.

12. Drummond MF, Sculpher MJ, Torrance GW, et al. Methods for the economic evaluation of health care programmes: Oxford University Press, 2005.

13. Beecham J, Knapp M. Client Service Receipt Inventory (CSRI). *Database of Instruments for Resource Use Management* 1992

14. NHS Improvement. NHS Reference costs 2017/2018 London: Department of Health; 2018. Available from: https://improvement.nhs.uk/resources/reference-costs.Accessed 1st September 2018.

For peer review only - http://bmjopen.bmj.com/site/about/guidelines.xhtml

15. Curtis L, Burns, Amanda. Unit Costs of Health & Social Care 2017. *PSSRU - University of Kent* 2017

16. British national formulary (BNF) 75: Joint Formulary Committee; 2018 Available from: www.medicinescomplete.com. Accessed 1st November 2018.

17. Prescription Cost Analysis - England, 2017 NHS Digital; 2018. Available from: https://digital.nhs.uk/data-and-information/publications/statistical/prescription-cost-analysis/prescription-cost-analysis-england-2017. Accessed 1st November 2018.

18. Bank of England Inflation Calculator 2018. Available from: https://www.bankofengland.co.uk/monetary-policy/inflation/inflation-calculator. Accessed 1st November 2018.

19. Williams A. The EuroQol instrument. EQ-5D concepts and methods: a developmental history: Springer 2005:1-17.

20. NICE. Position statement on use of the EQ-5D-5L valuation set, 2017.

21. Van Hout B, Janssen M, Feng Y-S, et al. Interim scoring for the EQ-5D-5L: mapping the EQ-5D-5L to EQ-5D-3L value sets. *Value in Health* 2012;15(5):708-15.

22. Billingham L, Abrams KR, Jones DR. Methods for the analysis of quality-of-life and survival data in health technology assessment. 1999

23. Willan AR, Briggs AH, Hoch JS. Regression methods for covariate adjustment and subgroup analysis for non-censored cost-effectiveness data. *Health Economics* 2004;13(5):461-75.

24. Gray AM, Clarke PM, Wolstenholme JL, et al. Applied methods of cost-effectiveness analysis in healthcare: Oxford University Press 2011.

25. Fenwick E, Claxton K, Sculpher M. Representing uncertainty: the role of cost-effectiveness acceptability curves. *Health Economics* 2001;10(8):779-87.

26. StataCorp. Stata Statistical Software: Release 15: College Station, TX: StataCorp LLC., 2017.

27. Pollock A, Farmer SE, Brady MC, et al. Interventions for improving upper limb function after stroke. *Cochrane Database of Systematic Reviews* 2014(11)

28. NICE. Developing NICE guidelines: the manual: chapter 4 - incorporating economic evaluation: National Institute for Health and Care Excellence Manchester, England, 2014.

29. Feeny D, Furlong W, Torrance GW, et al. Multiattribute and single-attribute utility functions for the health utilities index mark 3 system. *Medical Care* 2002;40(2):113-28.

For peer review only - http://bmjopen.bmj.com/site/about/guidelines.xhtml

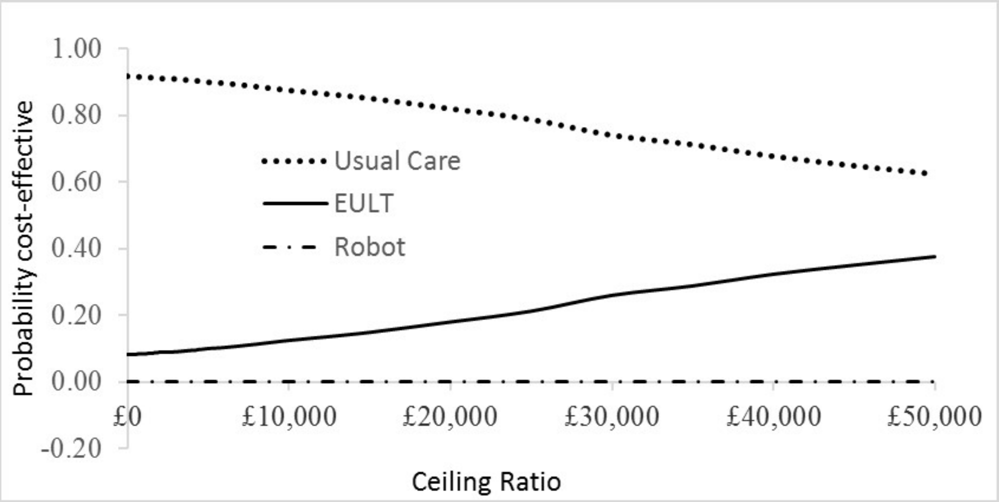

Caption : Figure 1 - Cost-effectiveness acceptability curve (base-case analysis) - adjusted bootstrapped replications for cost-effectiveness analysis

143x72mm (150 x 150 DPI)

CHEERS checklist—Items to include when reporting economic evaluations of health interventions

Section/item	Item No	Recommendation	Reported on page No/ line No
**Title and abstract**			
Title	1	Identify the study as an economic evaluation or use more specific terms such as "cost-effectiveness analysis", and describe the interventions compared.	Page 1/line 1-3
Abstract	2	Provide a structured summary of objectives, perspective, setting, methods (including study design and inputs), results (including base case and uncertainty analyses), and conclusions.	Page 2/line 24-57
**Introduction**			
Background and objectives	3	Provide an explicit statement of the broader context for the study.<br><br>Present the study question and its relevance for health policy or practice decisions.	Page 3-4/ line 63-80; Page 2 / Line 25-26
**Methods**			
Target population and subgroups	4	Describe characteristics of the base case population and subgroups analysed, including why they were chosen.	Page 2/ line 38-39; Page 7/ line 213-215
Setting and location	5	State relevant aspects of the system(s) in which the decision(s) need(s) to be made.	Page 2/ line 38-39, Page 14/ line 385-392
Study perspective	6	Describe the perspective of the study and relate this to the costs being evaluated.	Page 4/ line 117-118
Comparators	7	Describe the interventions or strategies being compared and state why they were chosen.	Page 2/ line 35-36; Page 3/ line 71-75
Time horizon	8	State the time horizon(s) over which costs and consequences are being evaluated and say why appropriate.	Page 2/ line 45-51; Page 4/5/ line 120-138 & 154-163; Page 7/ line 224-231
Discount rate	9	Report the choice of discount rate(s) used for costs and outcomes and say why appropriate.	Page 5 /line 128
Choice of health outcomes	10	Describe what outcomes were used as the measure(s) of benefit in the evaluation and their relevance for the type of analysis performed.	Page 2 /line 42-43; Page 5/line 154-163;
Measurement of effectiveness	11a	*Single study-based estimates:* Describe fully the design features of the single effectiveness study and why the single study was a sufficient source of clinical effectiveness data.	Page 5 /line 174-186;
	11b	*Synthesis-based estimates*: Describe fully the methods used for identification of included studies and synthesis of clinical effectiveness data.	Not applicable
Measurement and valuation of preference based outcomes	12	If applicable, describe the population and methods used to elicit preferences for outcomes.	Page 5/ line 154-163;
Estimating resources and costs	13a	*Single study-based economic evaluation:* Describe approaches used to estimate resource use associated with the alternative interventions. Describe primary or secondary research methods for valuing each resource item in terms of its unit cost. Describe any adjustments made to approximate to opportunity costs.	Page 4/5/ line 120-152; Page 6/ line 174-201

For peer review only

Section/item	Item No	Recommendation	Reported on page No/ line No
	13b	*Model-based economic evaluation:* Describe approaches and data sources used to estimate resource use associated with model health states. Describe primary or secondary research methods for valuing each resource item in terms of its unit cost. Describe any adjustments made to approximate to opportunity costs.	Page 7/ 223-236
Currency, price date, and conversion	14	Report the dates of the estimated resource quantities and unit costs. Describe methods for adjusting estimated unit costs to the year of reported costs if necessary. Describe methods for converting costs into a common currency base and the exchange rate.	Page 2/ line 41; Page 5/ line 151-152
Choice of model	15	Describe and give reasons for the specific type of decision-analytical model used. Providing a figure to show model structure is strongly recommended.	Page 7/ 223-236
Assumptions	16	Describe all structural or other assumptions underpinning the decision-analytical model.	Page 7/ line 225-231
Analytical methods	17	Describe all analytical methods supporting the evaluation. This could include methods for dealing with skewed, missing, or censored data; extrapolation methods; methods for pooling data; approaches to validate or make adjustments (such as half cycle corrections) to a model; and methods for handling population heterogeneity and uncertainty.	Page 6/ line 165-172; Page 6/ line 190-207; Page 7/ line 208-222
**Results**			
Study parameters	18	Report the values, ranges, references, and, if used, probability distributions for all parameters. Report reasons or sources for distributions used to represent uncertainty where appropriate. Providing a table to show the input values is strongly recommended.	Information available but not included in the manuscript
Incremental costs and outcomes	19	For each intervention, report mean values for the main categories of estimated costs and outcomes of interest, as well as mean differences between the comparator groups. If applicable, report incremental cost-effectiveness ratios.	Page 10/ table 2 & table 3; Page 12/ table 4; Page 13 figure 1;
Characterising uncertainty	20a	*Single study-based economic evaluation:* Describe the effects of sampling uncertainty for the estimated incremental cost and incremental effectiveness parameters, together with the impact of methodological assumptions (such as discount rate, study perspective).	Page 11/ line 288-307; Page 12/ table 4; Page 13/ line 323-338;
	20b	*Model-based economic evaluation:* Describe the effects on the results of uncertainty for all input parameters, and uncertainty related to the structure of the model and assumptions.	Page 11/ line 308-317; Page 12/13/ table 4;
Characterising heterogeneity	21	If applicable, report differences in costs, outcomes, or cost-effectiveness that can be explained by variations between subgroups of patients with different baseline characteristics or other observed variability in effects that are not reducible by more information.	Page 11 /line 295-302; Page 12/ table 4;

For peer review only

Section/item	Item No	Recommendation	Reported on page No/ line No
**Discussion**			
Study findings, limitations, generalisability, and current knowledge	22	Summarise key study findings and describe how they support the conclusions reached. Discuss limitations and the generalisability of the findings and how the findings fit with current knowledge.	Page 13/line 341-345<br>Page 14/ line 346-390<br>Page 15/line 391-414
**Other**			
Source of funding	23	Describe how the study was funded and the role of the funder in the identification, design, conduct, and reporting of the analysis. Describe other non-monetary sources of support.	Page 16/ line 433-436
Conflicts of interest	24	Describe any potential for conflict of interest of study contributors in accordance with journal policy. In the absence of a journal policy, we recommend authors comply with International Committee of Medical Journal Editors recommendations.	Page 16/ line 444-454

For peer review only
